# The oncolytic avian reovirus p17 protein triggers chaperone-mediated autophagy by modulating Hsp90 and the T-complex protein-1 ring complex chaperones and co-chaperones to activate the IKK/NF-κB signaling

**DOI:** 10.1128/jvi.01089-25

**Published:** 2025-12-03

**Authors:** Wei‐Ru Huang, Jyun-Yi Li, Tsai-Ling Liao, Lon-Fye Lye, Muhammad Munir, Hung-Jen Liu

**Affiliations:** 1 Institute of Molecular Biology, National Chung Hsing University 676969, Taichung, Taiwan; 2 The iEGG and Animal Biotechnology Center, National Chung Hsing University 34916, Taichung, Taiwan; 3 Department of Medical Research, Taichung Veterans General Hospital 40293, Taichung, Taiwan; 4 Rong Hsing Research Center for Translational Medicine, National Chung Hsing University 34916, Taichung, Taiwan; 5 Ph.D Program in Translational Medicine, National Chung Hsing University 34916, Taichung, Taiwan; 6 Department of Medical Research, Tungs' Taichung MetroHarbor Hospital 59084, Taichung, Taiwan; 7 Division of Biomedical and Life Sciences, Faculty of Health and Medicine, Lancaster University 151268, Lancaster, United Kingdom; 8 Department of Life Sciences, National Chung Hsing University 593984, Taichung, Taiwan; University of Michigan Medical School, Ann Arbor, Michigan, USA

**Keywords:** avian reovirus, p17, NF-κB, T-complex protein-1 ring complex, Hsp90/dc37 complex, TRiC/PhLP1 complex, chaperone-mediated autophagy

## Abstract

This study is the first to reveal that oncolytic avian reovirus (ARV) modulates the IKK/NF-κB signaling through the Hsp90-Cdc37, T-complex protein-1 ring complex (TRiC)/Hsc70, and TRiC-phosducin-like protein 1 (PhLP1) chaperone complexes, thereby inducing chaperone-mediated autophagy in mammalian and human cancer lines (referred to as Vero and A549). Specifically, the ARV p17 protein elevates the binding of co-chaperone PhLP1 to TRiC, thereby reducing the complex formation of TRiC-Hsc70 and leading to IκB degradation, suggesting that these two co-chaperones exhibit a competitive relationship. The p17 protein of ARV plays a crucial role in enhancing the formation of the TRiC/PhLP1 complex to protect viral proteins from ubiquitin-proteasome-mediated degradation. Interestingly, we found that ARV p17 enhances the interaction between the Hsp90/Cdc37 chaperone complex and IKK through activation of casein kinase 2 (CK2), which in turn activates NF-κB. Moreover, ARV p17 transcriptionally upregulates Beclin1 and increases the formation of Beclin1-PtdIns3K complex through the CK2-Hsp90/Cdc37 pathway. Immunofluorescence staining reveals that ARV p17 promotes the formation of GFP-LC3 puncta in both Vero and A549 cell lines, while a significant reduction in GFP-LC3 puncta was observed in CK2 and Hsp90 knockdown cells. Interestingly, *in situ* proximity ligation assay indicated that ARV p17 promotes the interaction between LC3-II and the cytosolic chaperonin complex 2, triggering chaperone-mediated autophagy. This study provides novel insights into ARV-modulated suppression of IκB by regulating co-chaperone PhLP1 and Hsc70 binding to TRiC and activation of the CK2/Hsp90/Cdc37/IKK/NF-κB pathway to induce chaperone-mediated autophagy.

**IMPORTANCE:**

This study reveals that the oncolytic avian reovirus (ARV) p17 activates chaperone-mediated autophagy in Vero and A549 cells by modulating the Hsp90/Cdc37, T-complex protein-1 ring complex (TRiC)/Hsc70, and TRiC/phosducin-like protein 1 (PhLP1) chaperone complexes. ARV p17 enhances PhLP1 binding to TRiC, reducing TRiC/Hsc70 complex formation, which promotes IκB degradation and activates the IKK/NF-κB pathway. Additionally, p17 increases casein kinase 2 (CK2)-mediated phosphorylation, strengthening the Hsp90/Cdc37/IKK interaction and transcriptionally upregulating Beclin1, forming the Beclin1/PtdIns3K complex to further induce autophagy. Immunofluorescence shows p17-induced GFP-LC3 puncta, which decreases upon CK2 and Hsp90 knockdown. *In situ* proximity ligation assay revealed that p17 promotes LC3-II and CCT2 interactions, confirming chaperone-mediated autophagy activation. This study provides novel insights into ARV-modulated suppression of IκB by modulating co-chaperone PhLP1 and Hsc70 binding to TRiC and activation of the CK2/Hsp90/Cdc37/IKK/NF-κB pathway to induce chaperone-mediated autophagy. This work expands our understanding of the role of ARV in regulating host cell autophagy pathways and viral replication. It also provides a new avenue for understanding viral modulation of host cellular processes in the context of oncolytic virotherapy.

## INTRODUCTION

NF-κB is a family of transcription factors involved in regulating the expression of various genes, including those related to immune responses, inflammation, cell proliferation, and apoptosis (1, 2). The activation of NF-κB is the result of multiple cellular signaling pathways and plays a crucial role in several viral infections. In the classical pathway, NF-κB activation is typically triggered by pro-inflammatory cytokines, including TNF-α and IL-1β (3, 4). This process involves the phosphorylation and degradation of IκB, allowing the NF-κB heterodimer to be released and translocated into the nucleus. On the other hand, the non-canonical pathway primarily involves the activation of NF-κB-inducing kinase and IKKα, which influence the processing of the p100 protein. This leads to the generation of the p52-RelB heterodimer (5, 6). A previous study has suggested that short wavelength UV light induces NF-κB activation through casein kinase 2 (CK2)-mediated phosphorylation and IκB degradation (7). The NF-κB complex, which remains inactive under normal conditions, can be activated by a range of stimuli such as viral and bacterial infections, proinflammatory cytokines, mitogens, growth factors, and stress-inducing factors (8, 9). Cellular autophagy is commonly initiated by the deactivation of mTORC1, leading to the activation of the ULK1 kinase complex (10). Moreover, autophagy regulation involves energy sensors like AMPK and several other signaling pathways, including the PI3K-Akt pathway (11, 12). Autophagy may generate a cellular environment that facilitates survival in various ways. In addition to its role in normal cellular metabolism and waste removal, autophagy is vital for providing protection in contexts such as pathogen infections and cellular stress (13). In most cells, four types of autophagy coexist: microautophagy, endosomal microautophagy, chaperone-mediated autophagy (CMA), and macroautophagy (14, 15). The NF-κB transcription factor family not only regulates cell survival, apoptosis, and inflammation but also contributes to the regulation of autophagy (16–18). IKK-NF-κB has been found to induce autophagy by directly stimulating the expression of key components of the autophagy machinery, including Beclin 1, ATG5, and LC3 (19, 20). Conversely, NF-κB can suppress autophagy by upregulating autophagy inhibitors such as Bcl-2 family proteins and the PTEN/mTOR signaling pathway (20, 21). The activation of NF-κB can stimulate the production of antiviral cytokines, such as IFN-β, which plays a key role in cellular antiviral defense. However, some viruses can exploit NF-κB-induced gene expression to enhance their own replication (8, 22). For example, the Tat protein of human immunodeficiency virus (HIV)-1 interacts directly with NF-κB, enhancing p65 transcriptional activity and thereby stimulating the viral LTR promoter (23). The X protein of the hepatitis B virus (HBV) triggers NF-κB activation by stimulating the IKK complex, promoting the expression and replication of viral genes (24). The NS1 protein of the influenza virus blocks NF-κB activation through inhibition of RIG-I and TRIM25, facilitating viral evasion from immune surveillance (25). ICP0, an E3 ligase found in herpes simplex virus 1, contains a RING finger domain at its N-terminus (26). This viral protein interacts with p50, tagging it for ubiquitination, which results in the suppression of NF-κB activity (27).

Chaperones in eukaryotic cells are classified into the heat shock protein family and the T-complex protein-1 ring complex (TRiC). Chaperone and co-chaperone complexes play a role in the refolding of nascent and misfolded proteins, as well as in promoting the degradation of ubiquitinated proteins through the proteasome (28). Various forms of stress, such as heat shock, oxidative stress, viral and bacterial infections, and chemical exposure, induce the expression of highly conserved heat shock proteins (29, 30). The heat shock proteins play a crucial role in regulating multiple signaling cascades, helping to preserve cellular homeostasis. Protein kinases are frequent client proteins of the Hsp90 chaperone, which oversees their formation, stability, and enzymatic activity (31). Recent studies indicate that Hsp70 and Hsp90 proteins modulate the IKK complex, which is a key activator of the NF-κB signaling pathway (32, 33). TRiC, also known as chaperonin-containing TCP-1 (CCT), is an essential eukaryotic chaperonin with a double-ring structure, each ring consisting of eight subunits (CCT1–8) (34, 35). The main function of TRiC is folding newly synthesized proteins with β-sheet topology, including tubulin, actin, and cellular proteins (36, 37). Recent findings suggested that TRiC plays a role in the folding of several viral proteins for different stages of the viral life cycle (38–40). Muscovy duck reovirus p10.8-induced cell cycle arrest and apoptosis depend on the involvement of Cdc20 and the TRiC chaperonins (40). In mammalian reovirus (MRV) replication, TRiC assists in folding the viral σ3 outer-capsid protein, allowing its assembly into viral particles (39). Another study highlights the crucial role of the CCT chaperonin in the processing and intracellular transport of HPV particles, as well as in subsequent entry during infection (38).

Avian reovirus (ARV) is a double-stranded RNA virus with a genome consisting of 10 discrete double-stranded RNA segments, encoding 5 non-structural proteins and 10 structural proteins (41, 42). Previous studies have demonstrated that the ARV p17 protein modulates multiple cellular signaling pathways (43–49). The ARV p17 protein shuttles between the nucleus and cytoplasm (45, 46), modulating multiple cellular signaling pathways (43, 47, 48) and leading to translation shutdown (48), cell cycle retardation (44, 49), and autophagosome formation (43, 47), all of which enhance viral replication. Recently, our team has demonstrated that the p17-modulated molecular chaperone Hsp90/Cdc37 enhances virus replication by protecting viral proteins (i.e., σA, σC, and σNS) from ubiquitin-proteasome degradation (50, 51). The current study aimed to explore the mechanisms by which the ARV p17 protein modulates the IKK/NF-κB signaling through the molecular chaperones TRiC and Hsp90 to induce chaperone-mediated autophagy.

## RESULTS

### The ARV p17 protein enhances the formation of the TRiC/PhLP1 complex to reduce the binding of co-chaperone Hsc70 to TRiC, leading to IκBα degradation

**Fig 1:**
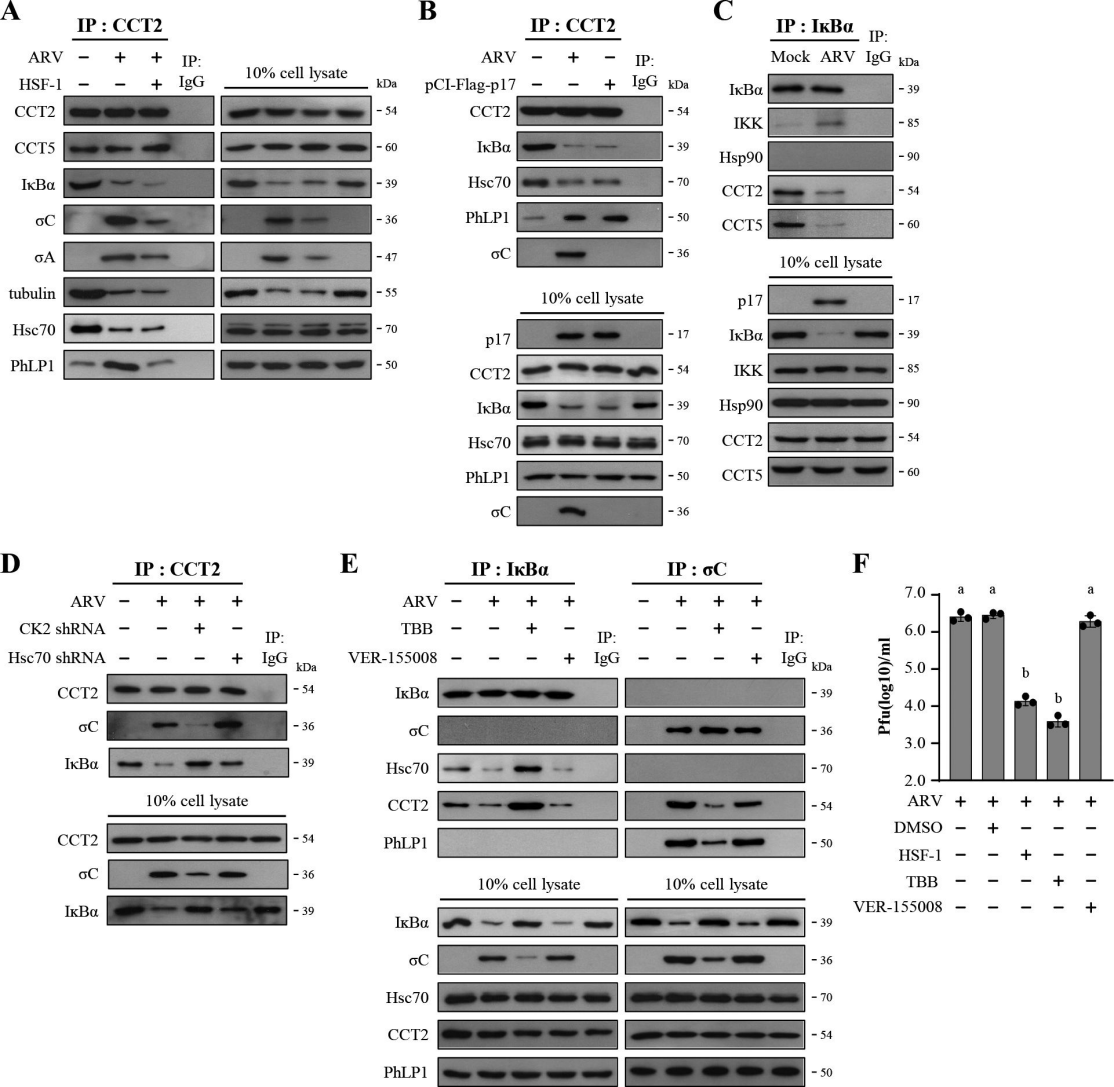
ARV regulates IκBα through the TRiC chaperone. (**A**) Vero cells were treated with TRiC inhibitor HSF-1 (40 µM) and then infected with ARV at an MOI of 10 for 24 h. In co-immunoprecipitation experiments, cell lysates were immunoprecipitated with CCT2 antibody and analyzed by Western blot assays with the indicated antibodies. (**B**) The binding of CCT2 to σC and IĸBα in ARV-infected or p17-transfected cells was examined. Cell lysates were immunoprecipitated with CCT2 and detected by Western blot assays with the indicated antibodies. (**C**) Vero cells were infected with ARV at an MOI of 10 for 24 h. In co-immunoprecipitation experiments, cell lysates were immunoprecipitated with IκBα antibody and analyzed by Western blot assays with the indicated antibodies. (**D**) Vero cells were transfected using the shRNAs for 6 h, followed by infection with ARV at an MOI of 10 for 24 h. In co-immunoprecipitation experiments, the binding of CCT2, ARV σC, and IκBα was examined in either ARV-infected or p17- and CK2 shRNA or Hsc70 shRNA-co-transfected Vero cells. Detection of protein expression was performed by Western blot assays with the indicated antibodies. (**E**) To study the role of co-chaperones in ARV-regulated NF-κB signaling, Vero cells were pretreated with either TBB (5 µM) or VER-155008 (5 µM) for 1 h followed by infection with ARV (10 MOI) for 24 h. Whole cell lysates were immunoprecipitated with anti-σC and anti-IĸBα antibodies and analyzed by Western blot assays with the indicated antibodies. Co-immunoprecipitation experiments of IκBα, σC, Hsc70, CCT2, and PhLP1 were performed as described above. IgG was used as a negative control. All experiments were conducted in three independent experiments. (**F**) Virus production was examined in ARV-infected or inhibitor-treated cells. The results were evaluated for statistical significance using Duncan’s multiple range test. The data were expressed as averages of three independent experiments. Similar letters denote no significance at *P* < 0.05. In this study, original blots and images are shown in Fig. S7.

Previously, we have demonstrated that ARV regulates chaperones Hsp90 and TRiC to stabilize viral proteins (i.e., σC, σA, σNS, and p17), which in turn facilitates viral replication and assembly (50, 51). Therefore, σC or σA proteins were the targets of detection in immunoprecipitation assays. In this study, we advanced our findings that ARV modulates IκBα/IKK signaling via the chaperones, resulting in the induction of autophagy and the promotion of viral replication. Initially, we identified that the TRiC/Hsc70 chaperone complex interacts with IκBα, suggesting that TRiC-Hsc70 protects IκBα from ubiquitin-proteasome-mediated degradation. As shown in Fig. 1A and B, co-immunoprecipitation results demonstrated that ARV infection and p17 transfection increase the formation of CCT2/PhLP1 complex to stabilize viral proteins and to reduce the amount of CCT2/Hsc70 complex. This resulted in reduced levels of IκBα binding to CCT2-Hsc70, leading to decreased levels of IκBα. The tubulin binding to CCT2 was also examined as a positive control (36). In contrast, inhibition of TRiC activity by HSF-1 (served as a negative control) resulted in decreased expression levels of IκBα and the viral proteins σC and σA (50, 51). Subsequently, we used CCT2 antibody immunoprecipitation to observe its interactions with different co-chaperones. According to the results, after 24 h of ARV infection or p17 transfection, immunoprecipitation with a CCT2 antibody revealed a significant decrease in binding with IκBα and Hsc70, whereas an increase in binding with PhLP1 was observed (Fig. 1B). These results demonstrate that ARV p17 modulates the interaction between co-chaperone PhLP1 and TRiC to stabilize viral proteins σC and σA (51). This finding also demonstrates that CCT2’s interactions with viral proteins and IκBα are mediated through a competitive mechanism. Furthermore, when performing immunoprecipitation using an IκBα antibody, a decrease in the interaction between TRiC and IκBα was observed in ARV-infected cells (Fig. 1C). These findings demonstrate that ARV enhances the expression levels of its viral proteins by modulating TRiC activity, accompanied by a decrease in the IκBα level. Previous reports suggested that phosphorylation of PhLP is mediated by the protein kinase CK2, and CCT works in concert with PhLP (52–54). To further study the roles of CK2, PhLP1, and Hsc70 in regulating IκBα and to investigate the interaction between CCT2 and the target proteins, both CK2 and Hsc70 were depleted using target-specific shRNAs, followed by ARV infection. The results revealed that ARV enhances the association of the σC protein of ARV with CCT2, leading to a reduction in the interaction between CCT2 and IκBα (Fig. 1D). In the CK2 shRNA-transfected cells, the interaction of ARV σC protein with CCT2 was reduced, consequently increasing the association between CCT2 and IκBα. Conversely, transfection with Hsc70 shRNA resulted in increased association of σC with CCT2 and reduced interaction with IκBα (Fig. 1D). Furthermore, by utilizing the Hsc70 and CK2 inhibitors, we identified that Hsc70 only interacted with IκBα and failed to bind to σC protein. This is the first report suggesting that ARV p17 inhibits IκBα binding to the TRiC-Hsc70 complex and modulates the TRiC/PhLP1 chaperone complex to stabilize viral proteins. Interestingly, PhLP1 was only observed in immunoprecipitation with the σC antibody, and its interaction with IκBα was not identified (Fig. 1E). This suggests that the TRiC/PhLP1 complex protects σC, but not IκBα, from ubiquitin-proteasome degradation. Immunoblots from panels A–E were quantitated by densitometry analysis (Fig. S1A through E). Correspondingly, the quantitative data of the average immunoblots showed the same trends. The findings demonstrate that CCT2 associates with σC and IκBα through different co-chaperones.

To further study whether TRiC, Hsp70, and CK2 affect virus replication, inhibition of TRiC by HSF-1 and CK2 by TBB was performed. Our results reveal that suppression of TRiC and CK2 significantly reduced virus yields (Fig. 1F), and no changes were observed in VER-155008 (Hsc70 inhibitor)-treated cells. To investigate whether inhibitors used in this study have deleterious effects on the cell, the viability of the cells was assessed by an MTT assay. As shown in Fig. S1F, cell activity was only slightly affected.

### ARV p17 enhances the phosphorylated form of CK2

**Fig 2:**
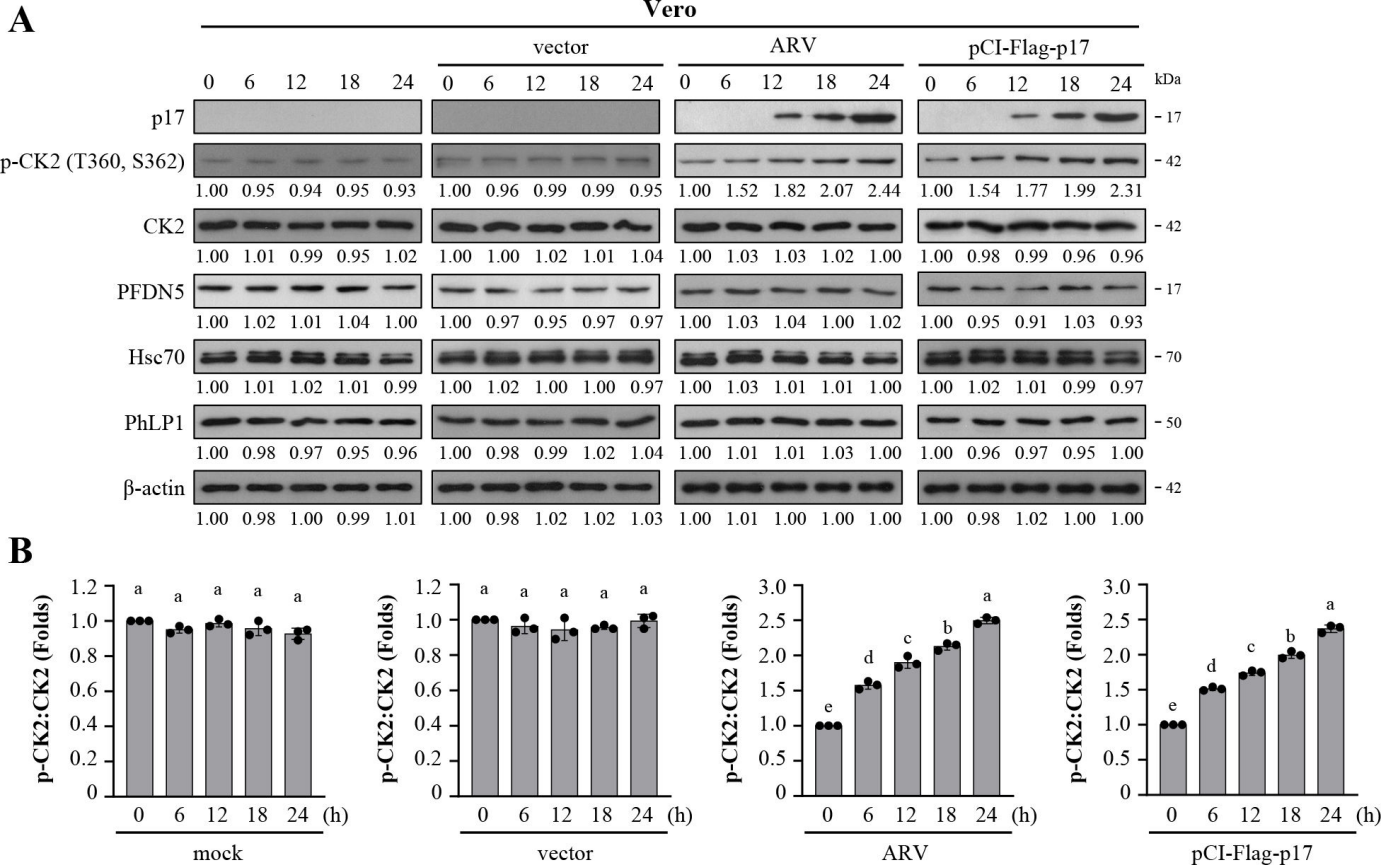
ARV p17 modulates the interaction between co-chaperones and TRiC without altering their protein expression levels. (**A**) To investigate whether the p17 protein modulates phosphorylation of CK2, protein levels of co-chaperones in ARV-infected and p17-transfected Vero cells were analyzed. The expression levels of p17, Hsp90, Cdc37, and p-Cdc37 in Vero cells infected with ARV at an MOI of 10 or transfected with the pCI-Flag-p17 plasmid were examined by Western blot assays. Vero cells were ARV-infected and pCI-Flag-p17-transfected at the indicated time points. The levels of the indicated proteins in the mock-control group (0 h) were considered onefold. The fold changes indicated below each lane were normalized against values for the mock-control group. Protein levels were normalized to those for β‐actin. Signals in all Western blots were quantified with ImageJ software. (**B**) Immunoblots showing the levels of p-CK2 in panel A were quantitated by densitometry analysis. The data were evaluated for statistical significance using Duncan’s multiple range test. The data were expressed as averages of three independent experiments.

Having shown that ARV infection and p17 transfection modulate the binding of PhLP1 to CCT2, thereby stabilizing viral proteins, we next aimed to investigate whether ARV infection and p17 transfection regulate the expression levels of co-chaperones of TRiC. Previously, it has been reported that CK2 regulates TRiC activity and promotes its interaction with PFDN5 to assist in protein folding (55). Following 24 h ARV post-infection or p17 transfection, whole cell lysates were collected for Western blot analysis to observe protein expression levels. The results indicate that ARV infection and p17 transfection did not alter the expression levels of co-chaperones Hsc70, PhLP1, and PFDN5 (Fig. 2A; Fig. S2); however, they resulted in an increase in the phosphorylation levels of p-CK2 in a time-dependent manner (Fig. 2A). Immunoblots from the levels of p-CK2 were quantitated by densitometry analysis (Fig. 2B). Correspondingly, the quantitative data of the average immunoblots showed the same trends.

### ARV induces chaperone-mediated autophagy in Vero cells

**Fig 3:**
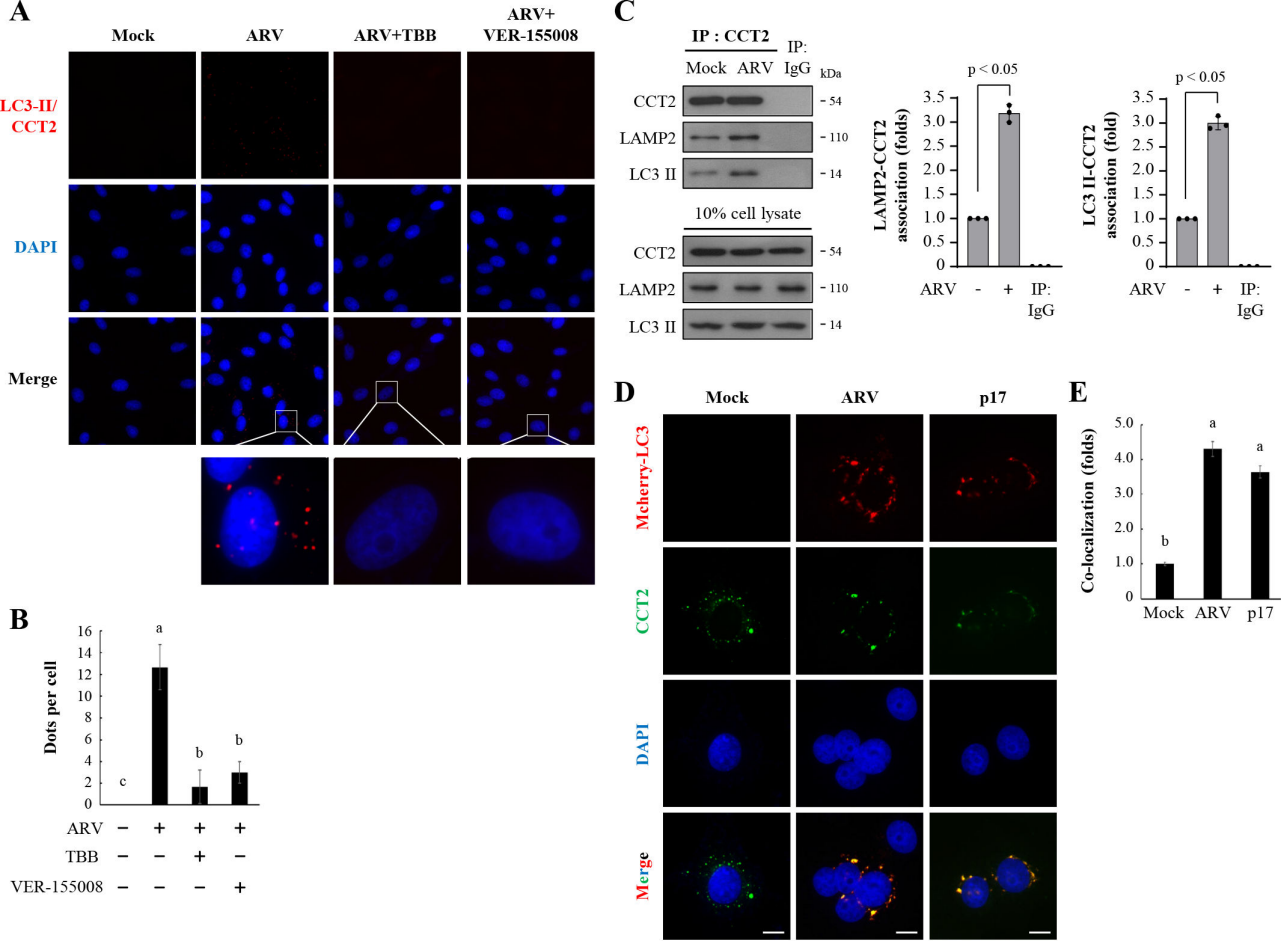
Proximity ligation assays for CCT2 and LC3-II detection in Vero cells. (**A**) To analyze interactions of CCT2 and LC3-II, Vero cells were detected using the commercial kit Duolink based on *in situ* PLA according to the manufacturer’s instructions. Enlarged images correspond to the region indicated by the white box in the merged image. Representative images are from three independent experiments. (**B**) The results (dots per cell) from panel A were quantitated. The results were evaluated for statistical significance using Duncan’s multiple range test. Data were collected from three independent experiments, with 15 randomly selected fields of view per experiment, and at least 200 cells were quantified in total. (**C**) To confirm ARV-mediated induction of CMA through CCT2, co-immunoprecipitation was performed to analyze the interactions among CCT2, LC3-II, and LAMP2. After 24 h of ARV infection, cellular proteins were collected for the co-immunoprecipitation assay. Whole cell lysates were immunoprecipitated with CCT2 antibody and analyzed by Western blot assays with the indicated antibodies. IgG was used as a negative control. Immunoblots from the left panel were quantitated by densitometry analysis. The data were evaluated for statistical significance using Student’s *t*-test. The data were expressed as averages of three independent experiments. *P* values < 0.05 were considered statistically significant. (**D**) Vero cells were transfected with the mCherry-LC3 plasmid for 6 h, followed by infection with ARV or transfection with pCI-Flag-p17 for 24 h. Vero cells were fixed and processed for immunofluorescence staining with DAPI (blue). Stained LC3 (red) and CCT2 (green) were observed under a fluorescence microscope. (**E**) The numbers of LC3 puncta were calculated from the results in panel D. Each value is the mean (with SD) from three independent experiments. The results were evaluated for statistical significance using Duncan’s multiple range test. Scale bars, 20 µm.

In most cells, three types of autophagy coexist, including microautophagy, endosomal microautophagy, chaperone-mediated autophagy, and macroautophagy (15). Interestingly, *in situ* proximity ligation assays (PLAs) indicate that ARV infection and p17 transfection promoted the interaction between LC3-II and CCT2, triggering chaperone-mediated autophagy (Fig. 3A). Additionally, the interaction of LC3-II with CCT2 was significantly reduced in cells treated with CK2 and Hsc70 inhibitors (Fig. 3A and B). According to existing reports, the formation of a complex involving several proteins is necessary to induce chaperone-mediated autophagy (14, 15, 56). Following 24 h of ARV infection or pCI-Flag-p17 transfection, an immunoprecipitation assay was performed using CCT2 antibodies. The results showed that ARV promotes the interaction of Lamp2 and LC3-II with CCT2 (Fig. 3C) but reduces the amount of IκBα binding to CCT2. Additionally, immunofluorescence staining confirmed the findings, demonstrating that ARV infection and p17 transfection enhance the interaction between LC3-II and CCT2, which triggers chaperone-mediated autophagy (Fig. 3D and E).

### ARV p17 acts as an NF-κB activator

**Fig 4:**
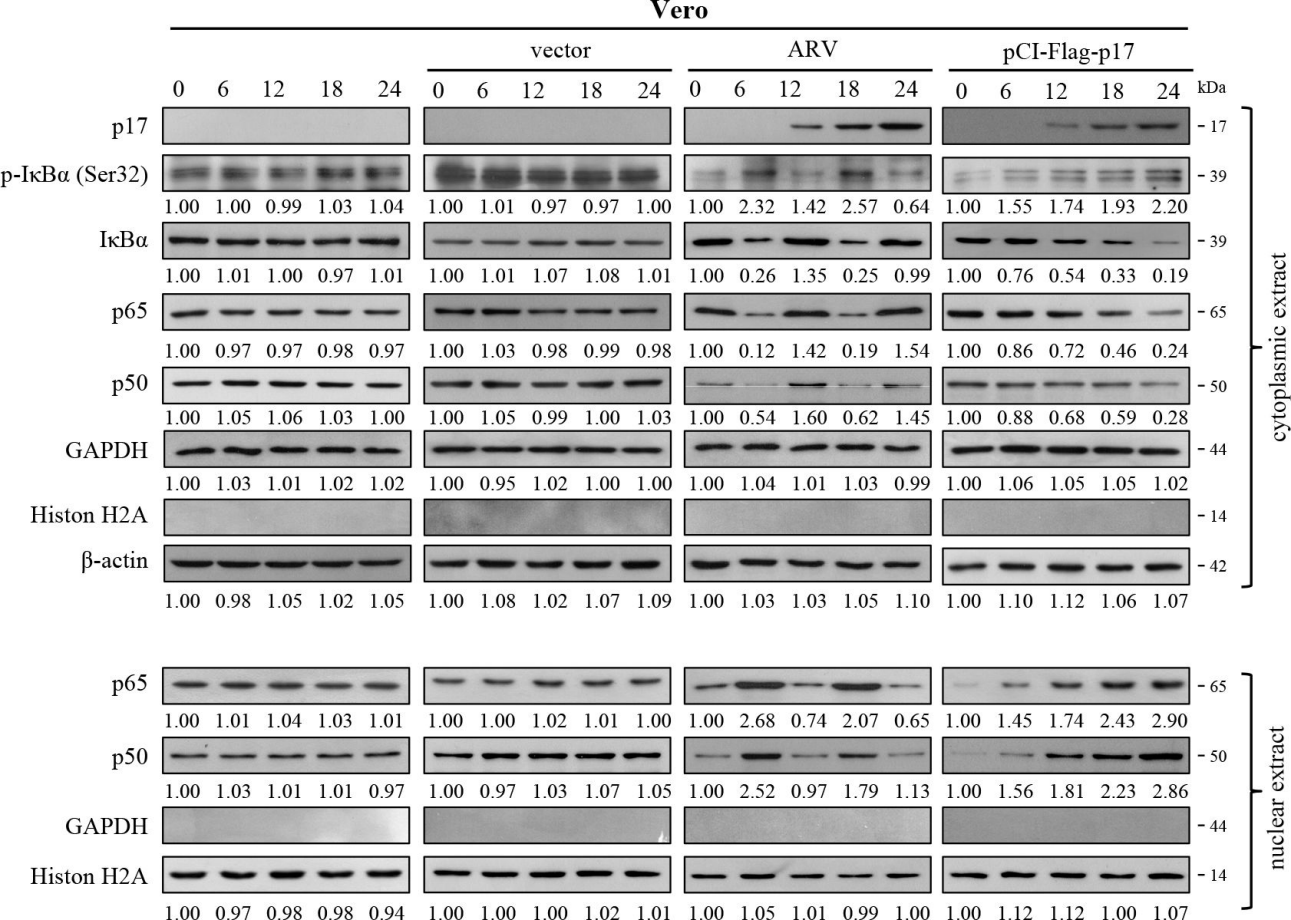
The p17 protein of ARV acts as an NF-κB activator. The levels of p-IκB, IκB, p65, and p50 in mock control, pCI-Flag vector, ARV-infected, and pCI-Flag-p17-transfected cells were examined. Cytoplasmic and nuclear protein extractions were carried out using the compartmental protein extraction. The cytoplasmic extracts and nuclear extracts were detected at the indicated time points for Western blot assays. The protein levels were normalized to that for β-actin or histone 2A. Numbers below each lane are percentages of the control level of a specific protein in the mock treatment.

To investigate whether ARV infection and p17 transfection activate NF-κB at different time points in Vero cells, whole cell lysates were collected for cytoplasmic and nuclear protein isolation, followed by Western blot analysis to evaluate the levels of p-IκBα and localization of p65 and p50 within the nucleus. The results demonstrate that the phosphorylation of IκBα (Ser32) is increased by both ARV-infected and p17-transfected cells. This, in turn, reduced the cytoplasmic levels of p65 and p50 and increased the levels of p65 and p50 in the nucleus, suggesting that ARV p17 suppresses IκBα to promote p65 and p50 translocation into the nucleus (Fig. 4), thereby activating NF-κB. Interestingly, the levels of p-IκBα exhibited two distinct peaks in ARV-infected cells, occurring at 6 and 18 h post-infection. The discrepancy in NF-κB nuclear translocation between ARV infection and p17 transfection likely arises from fundamental biological differences. ARV infection encompasses the complete viral replication cycle, during which different stages can differentially modulate host signaling pathways. In contrast, p17 transfection represents a simplified model in which only a single viral protein is expressed, resulting in a more uniform and direct modulation of NF-κB signaling. Immunoblots from Fig. 4 were quantitated by densitometry analysis (Fig. S3B). Correspondingly, the quantitative data of the average immunoblots showed the same trends. The finding corresponds to our previous report, confirming the increased levels of LC3-II in ARV-infected cells at 9 and 24 h post-infection (43).

### The ARV p17 protein modulates the protection of IKK from ubiquitin-proteasome-mediated degradation through the CK2/Hsp90/Cdc37 signaling and enhances IκB degradation

**Fig 5:**
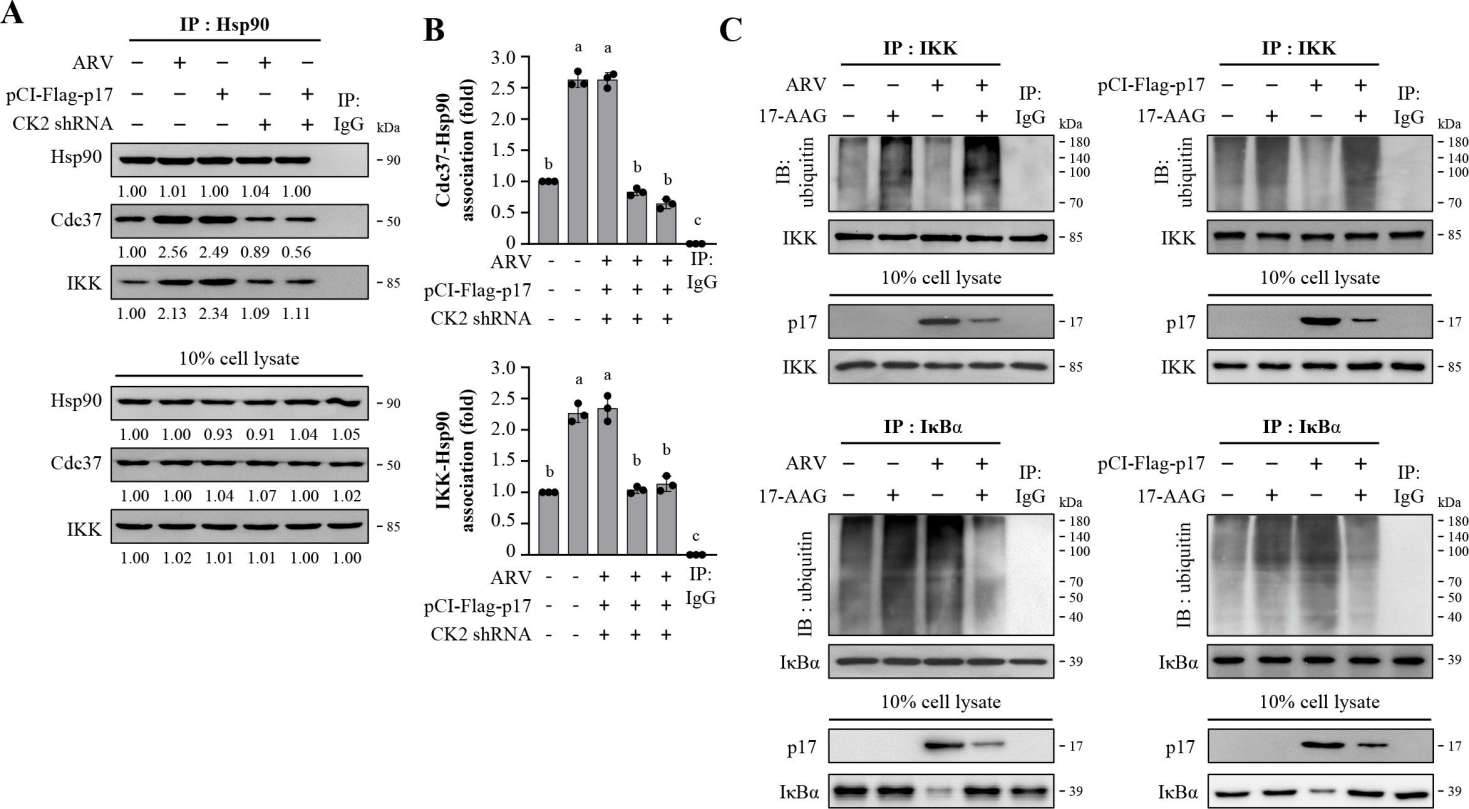
The p17 protein of ARV protects IKK from polyubiquitination and enhances IκB degradation. (**A**) Vero cells were transfected with CK2 shRNA for 6 h, followed by infection with ARV at an MOI of 10 for 18 h. The binding of Hsp90 to Cdc37 and IKK in ARV-infected or p17-transfected cells was examined in the presence or absence of CK2 shRNA. Cell lysates were immunoprecipitated with the Hsp90 antibody and detected with the indicated antibodies. (**B**) Immunoblots from panel A were quantitated by densitometry analysis. The data were evaluated for statistical significance using Duncan’s multiple range test. The data were expressed as averages of three independent experiments. (**C**) To investigate whether ARV p17-induced Hsp90 protects IKK or IκB proteins from ubiquitin-proteasome-mediated degradation, Vero cells were pretreated with 17-AAG (5 µM) for 1 h, followed by infection with ARV (10 MOI) or transfection with pCI-Flag-p17 for 18 h. Cell lysates were immunoprecipitated with IKK or IκB antibodies, and the interaction of ubiquitin with IKK and IκB was examined. The amounts of IKK and IκB polyubiquitylation were examined by Western blot assays. Rabbit IgG served as a negative control. Similar results were obtained in three independent experiments.

Having shown that the phosphorylation of IκBα (Ser32) increased in the ARV-infected or p17-transfected cells, we next wanted to confirm whether p17 modulates the CK2/Hsp90/Cdc37 signaling to protect IKK from ubiquitin-proteasome-mediated degradation. Vero cells were transfected with CK2 shRNA for 6 h, followed by infection with ARV at an MOI of 10 for 18 h. After 18 h, whole cell lysates were collected for immunoprecipitation using an Hsp90 antibody to analyze protein interactions. As shown in Fig. 5A, both ARV infection and p17 transfection significantly enhanced the interaction among Hsp90, Cdc37, and IKK. The amount of IKK binding to the Hsp90-Cdc37 complex was significantly reduced in CK2 knockdown cells. Immunoblots from Fig. 5A were quantitated by densitometry analysis (Fig. 5B). To validate the effectiveness of the shRNAs used in this study, we evaluated their knockdown efficiency by Western blot analysis. As shown in Fig. S1G, CK2 was depleted. To investigate whether shRNAs used in this study have deleterious effects on the cell, the viability of the cells was assessed by an MTT assay. As shown in Fig. S1F, cell viability was only slightly affected.

To confirm the interaction between IKK, Hsp90, and Cdc37 in preventing protein degradation, the ubiquitination of IKK and IκBα was analyzed. Vero cells were pretreated with 17-AAG, followed by infection with ARV or transfection with pCI-Flag-p17 for 18 h. Cell lysates were collected, and immunoprecipitation was performed using IKK and IκBα antibodies, respectively, followed by Western blot analysis for IKK and IκBα ubiquitination. The results revealed that ARV infection or p17 transfection led to a reduction in IKK ubiquitination, accompanied by an increase in IκBα ubiquitination (Fig. 5C). It was confirmed that ARV p17 facilitates the translocation of NF-κB into the nucleus (Fig. 4). Immunoprecipitation results indicate that ARV p17 was unable to significantly reduce IKK ubiquitination in Hsp90 inhibitor (17-AAG)-treated cells, and a reduction in IκBα ubiquitination was observed (Fig. 5C). These findings suggest that ARV p17 modulates protection of IKK from ubiquitin-proteasome-mediated degradation through the CK2/Hsp90/Cdc37 signaling.

### The ARV p17 protein induces autophagy through the Hsp90/Cdc37/IKK/NF-κB pathway

**Fig 6:**
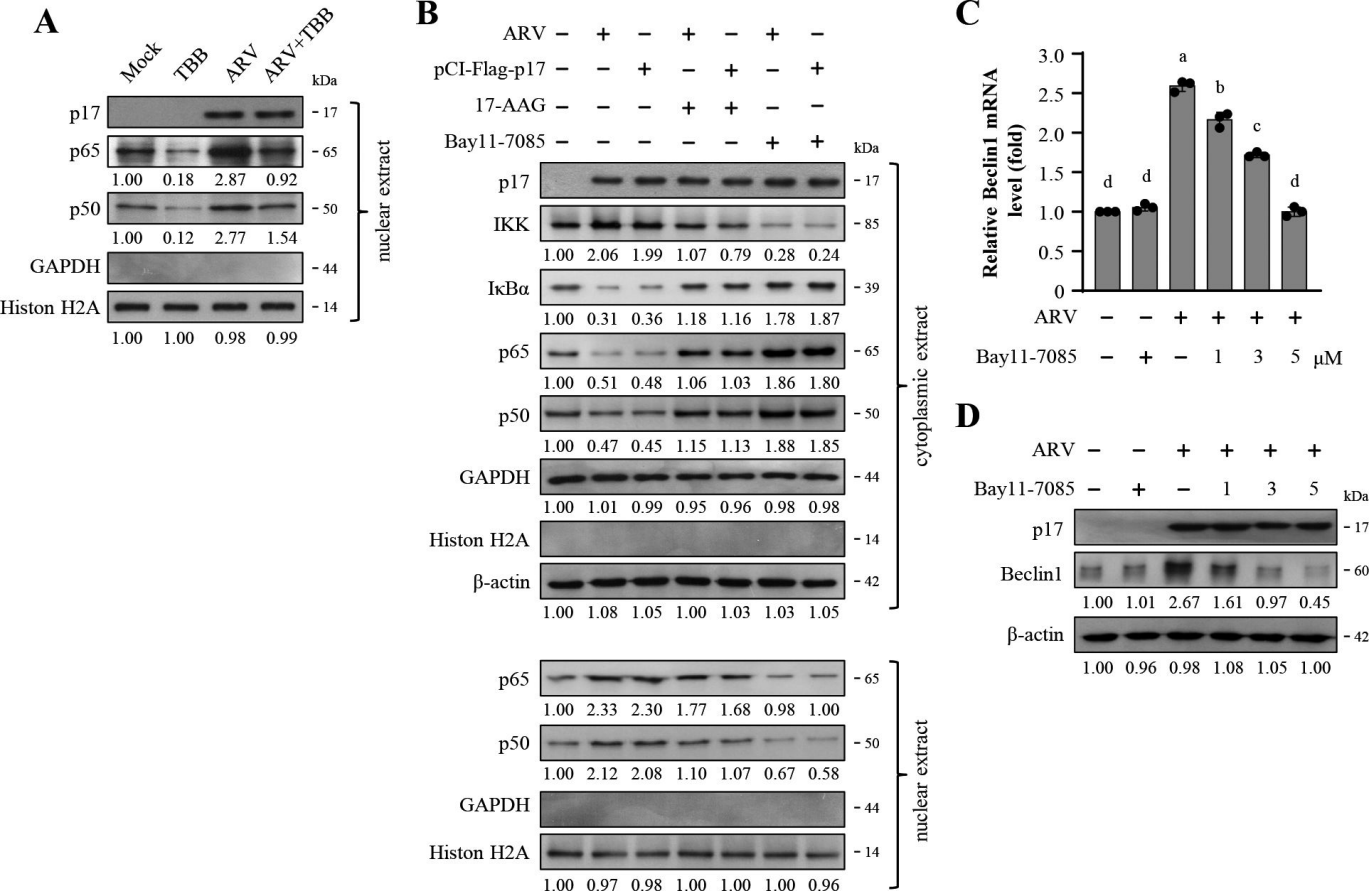
ARV p17-induced autophagy through the Hsp90/NF-κB pathway. (**A**) The expression level of p65 and p50 in ARV-infected cells was examined in the presence or absence of TBB (5 µM). Nuclear extracts from ARV-infected and mock-infected Vero cells were collected for Western blots. The protein levels were normalized to that for histone 2A. The levels of indicated proteins at mock were considered onefold. (**B**) To study the role of Hsp90 in p17-mediated regulation of the NF-κB pathway, Vero cells were pretreated with either 17-AAG (5 µM) or Bay11-7085 (5 µM) for 1 h, followed by infection with ARV (10 MOI) or transfection with pCI-Flag-p17 for 18 h. The cytoplasmic lysates and nuclear extracts were collected for Western blots. The levels of indicated proteins in the mock control or at 0 h were considered onefold. (**C**) The mRNA levels of Beclin1 in the Vero cells were examined by semi-quantitative RT-PCR in ARV-infected and mock-infected cells in the presence or absence of the different concentrations of Bay11-7085. After electrophoretic separation in an agarose gel, PCR products were stained with ethidium bromide. The data were evaluated for statistical significance using Duncan’s multiple range test. The graph shown represents the mean ± SE calculated from three independent experiments. (**D**) The expression level of Beclin1 in ARV-infected and mock-infected cells was examined in the presence or absence of Bay11-7085. Whole cell lysates were harvested for Western blots.

Having demonstrated that ARVp17 protein protects IKK from ubiquitin-proteasome-mediated degradation through the CK2/Hsp90/Cdc37 signaling, we next wanted to investigate the role of CK2 in nuclear translocation of NF-κB. ARV-infected cells were treated with the CK2 inhibitor (i.e., TBB). After 18 h of incubation, whole cell lysates were harvested for analyzing the nuclear translocation of p65 and p50 proteins. The data indicate that ARV enhances the nuclear translocation of p65 and p50 subunits of NF-κB (Fig. 6A; Fig. S4A). However, in the presence of TBB, which inhibits CK2, the nuclear translocation of p65 and p50 was significantly reduced (Fig. 6A), demonstrating that ARV mediates NF-κB translocation via the CK2/Hsp90/Cdc37 pathway. Furthermore, to investigate whether Hsp90 modulates NF-κB translocation, cells were infected with ARV or transfected with pCI-Flag-p17, followed by treatment with the Hsp90 inhibitor 17-AAG and the NF-κB inhibitor Bay11-7085. After 18 h, cytoplasmic and nuclear proteins were collected to analyze the translocation of p65 and p50 proteins. The results showed that in the ARV-infected or p17-transfected cells, there was an increase in cytoplasmic levels of IKK and a reduction in the levels of IĸBα (Fig. 6B; Fig. S4B). When Vero cells were treated with the Hsp90 inhibitor 17-AAG, the ARV- and p17-mediated translocation was significantly diminished in 17-AAG and Bay11-7085-treated cells (Fig. 6B; Fig. S4B). The findings demonstrate that ARV infection and p17 protein modulate the IKK and IκBα levels through the CK2/Hsp90/Cdc37 pathway, thereby regulating the translocation of p65 and p50 subunits of NF-κB. It has previously been shown that translocation of NF-κB into the nucleus regulates the expression of various genes, promoting survival, proliferation, inflammation, immune regulation, and autophagy (4). Therefore, we further investigated whether ARV regulates the expression of the key autophagy protein (i.e., Beclin1) through the NF-κB pathway. Following ARV infection, cells were treated with different concentrations of the Bay11-7085 inhibitor. After 18 h, cell lysates were collected for analysis through RT-PCR and Western blot. As shown in Fig. 6C and D and Fig. S4C and D, the results demonstrated that ARV dramatically enhances the mRNA and protein levels of Beclin1, suggesting that ARV transcriptionally upregulates Beclin1. However, upon the addition of Bay11-7085, the mRNA and protein levels of Beclin1 were significantly reduced, confirming that ARV elevates the expression level of Beclin1 via the NF-κB pathway.

### ARV p17 enhances the PtdIns3K-Beclin1 complex formation, leading to the induction of autophagosome formation

**Fig 7:**
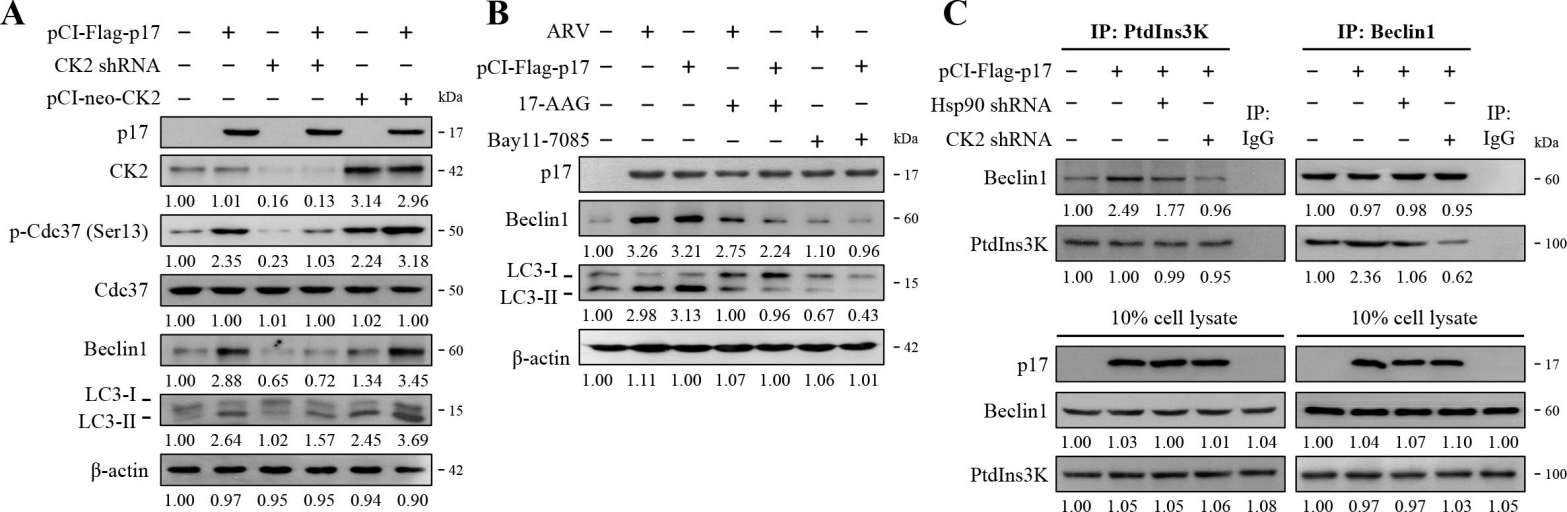
The p17 protein of ARV enhances the PtdIns3K/Beclin1 complex, leading to the induction of autophagosome formation. (**A**) To confirm whether CK2 is the upstream signaling that regulates Cdc37, Beclin1, and LC3, the protein levels were examined in CK2 knockdown and CK2 overexpression in p17-transfected Vero cells. The protein levels were normalized to that for β-actin. The activation and inactivation folds indicated below each lane were normalized against the mock control. (**B**) Vero cells were pretreated with either 17-AAG (5 µM) or Bay11-7085 (5 µM) for 1 h, followed by infection with ARV (10 MOI) or transfection with pCI-Flag-p17 for 18 h. Whole cell lysates were collected for Western blots with the indicated antibodies. The levels of indicated proteins at mock were considered onefold. (**C**) ARV p17 facilitates autophagosome formation by promoting the PtdIns3K/Beclin1 complex via the CK2/Hsp90 pathway. In reciprocal co-immunoprecipitation experiments, the binding of PtdIns3K and Beclin1 was examined in either p17-transfected or p17 and Hsp90 or CK2 shRNA-co-transfected Vero cells and detected with the indicated antibody. The activation and inactivation folds indicated below each lane were normalized against that at the mock control.

After demonstrating that the ARV p17 protein modulates NF-κB translocation through the CK2/Hsp90/Cdc37 pathway, we next explored whether this pathway is also involved in the formation of the PtdIns3K-Beclin1 complex. Vero cells were transfected with the pCI-Flag-p17 plasmid for 6 h, followed by transfection of CK2 shRNA or the pCI-neo-CK2 plasmid. After 18 h of co-transfection, cell lysates were collected for Western blot analysis. In the p17-transfected cells, the levels of p-Cdc37 (Ser13), Beclin1, and LC3-II showed significant increases (Fig. 7A; Fig. S5A), while these enhancements were notably suppressed in the CK2 shRNA co-transfected cells (Fig. 7A). Interestingly, when pCI-Flag-p17 and pCI-neo-CK2 plasmids were co-transfected, the expression levels of p-Cdc37 (Ser13), Beclin1, and LC3-II were significantly increased (Fig. 7A; Fig. S5A), suggesting that ARV p17 induces autophagy via the CK2/Hsp90/Cdc37 pathway. However, when the Hsp90 inhibitor 17-AAG or the NF-κB inhibitor Bay11-7085 was used, ARV p17 failed to increase the Beclin1 and LC3-II expression levels (Fig. 7B; Fig. S5B), further confirming that the ARV p17 protein enhances autophagy through the CK2/Hsp90/Cdc37 pathway. Cellular autophagy appears through the assembly of multiple proteins into a complex that drives the autophagic formation (15). Thus, we utilized immunoprecipitation to investigate the status of the autophagy protein complex in cells after ARV infection. As shown in Fig. 7C and Fig. S5C, while using class III PI3K or Beclin1 as immunoprecipitation antibodies, the formation of the PtdIns3K and Beclin1 complex was significantly increased in the p17-transfected cells. However, upon co-transfection of CK2 or Hsp90 shRNA, the interaction between PtdIns3K and Beclin1 was decreased, further supporting the idea that p17 induces autophagy formation through the CK2/Hsp90 pathway. To validate the effectiveness of the shRNAs used in this study, we evaluated their knockdown efficiency by Western blot analysis. As shown in Fig. S1G, CK2 and Hsp90 were depleted. To investigate whether shRNAs used in this study have deleterious effects on the cell, cell viability was assessed by an MTT assay. As shown in Fig. S1F, cell viability was only slightly affected.

### The ARV p17 protein induces autophagosome formation through the CK2/Hsp90 pathway

**Fig 8:**
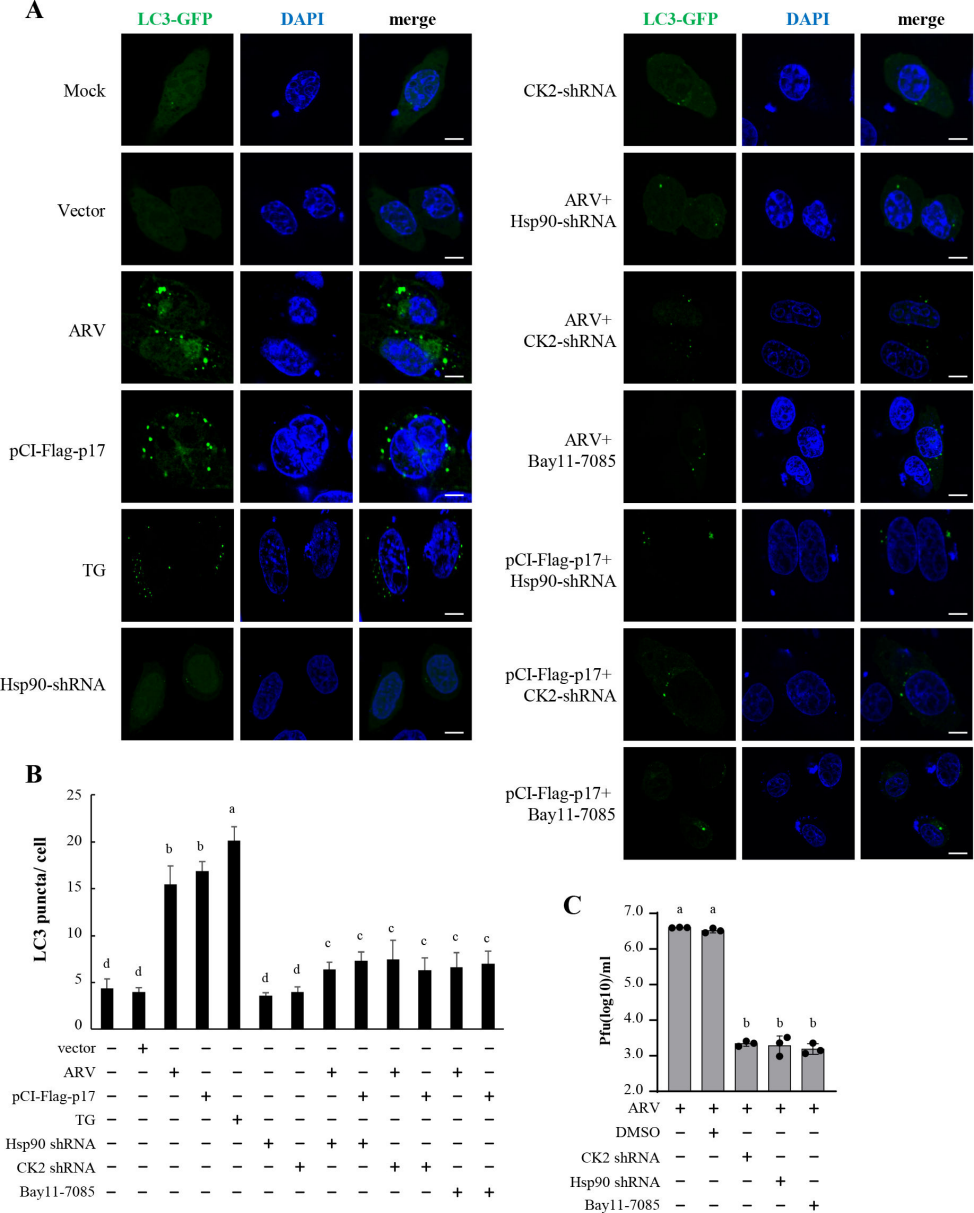
ARV p17 promotes autophagosome formation in Vero cells. (**A**) Vero cells were transfected with the mCherry-LC3 plasmid and co-transfected with Hsp90 or CK2 shRNAs for 6 h, followed by the infection of ARV at an MOI of 10 for 18 h or pCI-Flag-p17 transfection for 24 h. Cells were then fixed and processed for immunofluorescence staining with DAPI. Cells were pretreated with TG for 2 h before the mCherry-LC3 plasmid transfection (positive controls). mCherry-LC3 plasmid was applied to observe LC3 puncta under a fluorescence microscope. Scale bars, 10 µm. (**B**) Quantitation results from panel A represent mean LC3-GFP puncta per cell. Data were collected from three independent experiments, with 17 randomly selected fields of view per experiment, and at least 100 cells were quantified in total. All image analyses were performed in a blind manner. The results were evaluated for statistical significance using Duncan’s multiple range test. (**C**) Virus production was examined in ARV-infected, shRNA-transfected, or inhibitor-treated cells. The results were evaluated for statistical significance using Duncan’s multiple range test. The data were expressed as averages of three independent experiments.

To analyze autophagosome formation, cells were transfected with the mCherry-LC3 plasmid to express the GFP-LC3 fusion protein, acting as a fluorescent indicator for autophagosomes. LC3-I shows a diffuse staining pattern in the cytoplasm, while LC3-II appears as distinct punctate staining (57). In both ARV-infected and p17-transfected Vero cells, a substantial increase in LC3-GFP puncta was observed (Fig. 8A). However, co-transfection with Hsp90 or CK2 shRNA led to a marked reduction in LC3-GFP puncta (Fig. 8A). A comparable trend was noted in thapsigargin (TG)-treated Vero cells compared to the mock control. Correspondingly, the quantitative data of the average LC3-GFP puncta per cell showed the same trends (Fig. 8B).

### ARV p17-induced autophagy through the Hsp90/NF-κB pathway in A549 cancer cells

**Fig 9:**
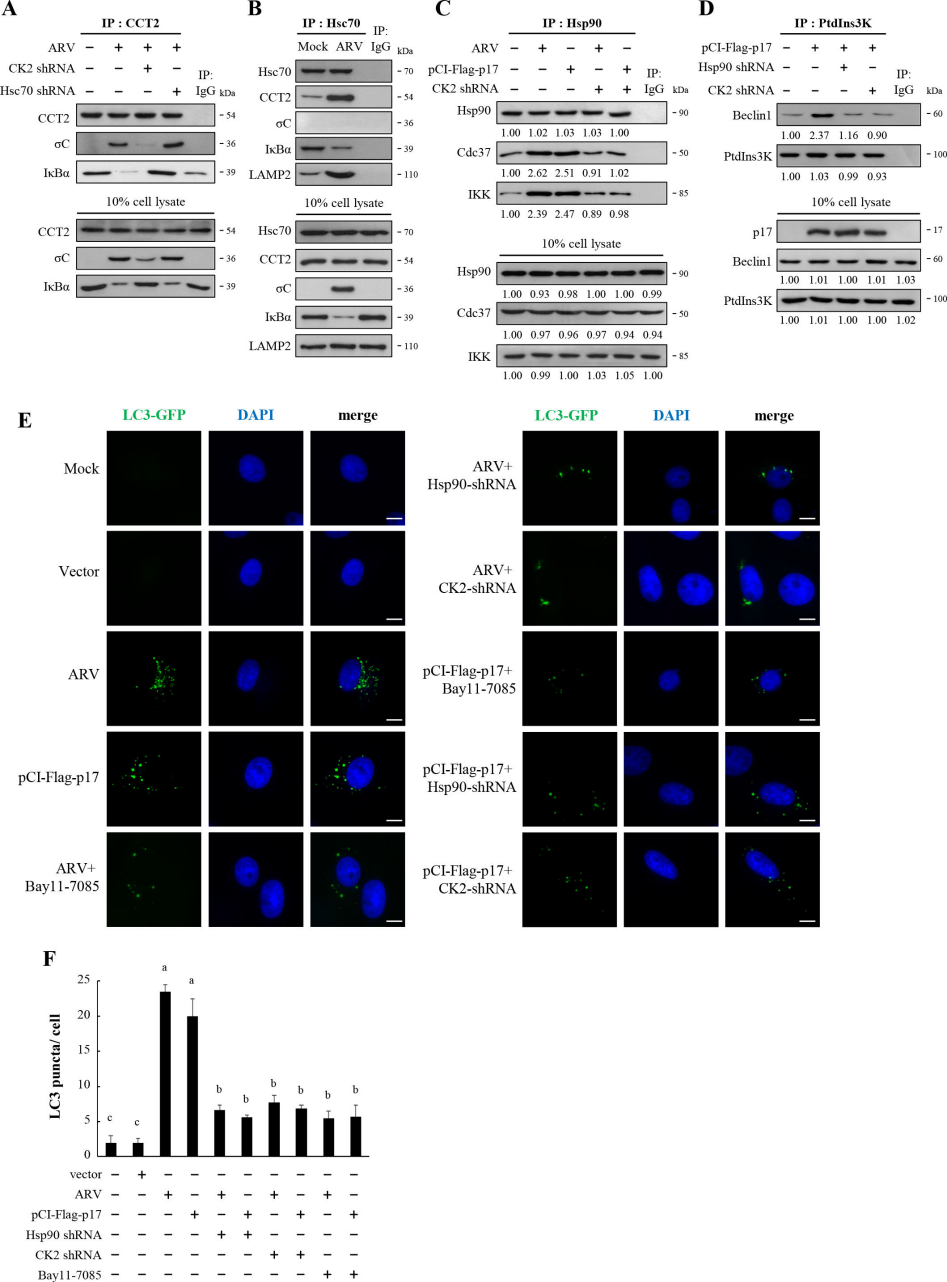
ARV p17 induced autophagy through the Hsp90/NF-κB pathway in A549 cancer cells. (**A**) A549 cells were transfected with the CK2 or Hsc70 shRNAs for 6 h, followed by infection with ARV at an MOI of 10 for 24 h. In co-immunoprecipitation experiments, the binding of CCT2, ARV σC, and IκBα was examined and detected by Western blot assays with the indicated antibodies. (**B**) A549 cells were infected with ARV at an MOI of 10 for 24 h. Cell lysates were collected for immunoprecipitation with the Hsc70 antibody and analyzed by Western blot assays with the indicated antibodies. (**C**) The binding of Hsp90 to Cdc37 and IKK in ARV-infected or p17-transfected cells was examined in the presence or absence of CK2 shRNA. Cell lysates were immunoprecipitated with Hsp90 and detected with the indicated antibodies. (**D**) In the co-immunoprecipitation experiment, the binding of class III PI3K was examined in either p17-transfected or p17 and Hsp90 shRNA or CK2 shRNA-cotransfected A549 cells and detected with the indicated antibodies. The activation and inactivation folds indicated below each lane were normalized against that at the mock control. (**E**) A549 cells were transfected with the LC3-GFP plasmid and co-transfected with Hsp90 or CK2 shRNAs for 6 h, followed by the infection of ARV with an MOI of 10 for 18 h or pCI-Flag-p17 transfection for 24 h. Cells were then fixed and processed for immunofluorescence staining with DAPI. The LC3-GFP was observed under a fluorescence microscope. Each experiment was performed at least three times. Scale bars, 20 µm. (**F**) The numbers of LC3 puncta were calculated from the results shown in panel E. Data were collected from three independent experiments, with 17 randomly selected fields of view per experiment, and at least 100 cells were quantified in total. All image analyses were performed in a blind manner. Each value represents mean ± SD of three independent experiments. The results were evaluated for statistical significance using Duncan’s multiple range test.

We have confirmed that ARV p17 induces autophagy by modulating Beclin 1/PtdIns3K complexes through the CK2/Hsp90/NF-κB pathway in Vero cells. Previous studies have identified ARV as an oncolytic virus (49), leading us to further investigate whether ARV p17 exhibits similar functions in A549 cancer cell lines. A549 cells were transfected with CK2 or Hsc70 shRNAs for 6 h, followed by infection with ARV at an MOI of 10 for 24 h. After 24 h, cell lysates were collected, and CCT2 immunoprecipitation was performed to analyze protein interactions. The data revealed that ARV facilitates the binding of ARV σC protein to CCT2, resulting in a decreased interaction between CCT2 and IκBα in A549 cancer cells (Fig. 9A; Fig. S6A). Upon transfection with CK2 shRNA, the ARV σC-CCT2 interaction was weakened, resulting in a higher association between CCT2 and IκBα. In contrast, silencing of Hsc70 using Hsc70 shRNA enhanced the binding of σC with CCT2 while reducing its interaction with IκBα (Fig. 9A; Fig. S6A). After ARV infection of A549 cells for 24 h, cell lysates were collected and subjected to immunoprecipitation with an Hsc70 antibody, revealing that Hsc70 interacted exclusively with IκBα and showed no binding to σC (Fig. 9B; Fig. S6B). Moreover, A549 cells were infected with ARV or transfected with pCI-Flag-p17 and then co-transfected with CK2 shRNA. Twenty-four hours later, cell lysates were harvested, and immunoprecipitation was carried out with an Hsp90 antibody to analyze the interactions between proteins. The findings demonstrate that both ARV infection and p17 transfection substantially promoted the interaction between Hsp90, Cdc37, and IKK, but this interaction was notably reduced upon CK2 shRNA co-transfection (Fig. 9C; Fig. S6C). Next, we investigated whether ARV induces autophagy in A549 cancer cells. Immunoprecipitation was used to analyze the state of the autophagy protein complex in A549 cancer cells following ARV infection. Using PtdIns3K as the immunoprecipitation antibody, immunoprecipitation assays revealed that p17-transfected cells substantially increased the formation of the PtdIns3K and Beclin1 complex (Fig. 9D; Fig. S6D). However, co-transfection with CK2 or Hsp90 shRNA reduced this interaction (Fig. 9D), suggesting that ARV p17 promotes autophagy of A549 cancer cells through the CK2/Hsp90 pathway. The immunofluorescence results in Fig. 9E demonstrate that LC3-GFP puncta increased substantially in both ARV-infected and p17-transfected A549 cells. However, co-transfection with Hsp90 or CK2 shRNA notably reduced the LC3-GFP puncta (Fig. 9E).

## DISCUSSION

Viruses depend on host cells for survival, utilizing cellular mechanisms for gene replication and viral particle assembly. Despite notable advancements in research on viral regulation of host cell mechanisms, numerous issues are still unanswered. Previously, we have demonstrated that ARV facilitates viral replication by modulating cellular translation, cell cycle, and autophagy (43, 44, 47, 49). Recent studies indicated that ARV utilizes TRiC and Hsp90 chaperone systems to support viral protein synthesis and particle assembly (50, 51). This study, for the first time, demonstrates that ARV modulates the suppression of IκBα by regulating the co-chaperones PhLP1 and Hsc70 and activates the IKK/NF-κB signaling through TRiC and Hsp90 chaperones to induce chaperone-mediated autophagy, benefiting viral replication. The function of CCT is modulated by prefoldin, PhLPs, and Hsc70 to ensure precise control over the protein folding process (36). However, the folding of multidomain proteins often requires the coordinated action of multiple chaperone classes, which collaborate to provide a safeguarded folding pathway. This indicates that CCT is tightly regulated by co-chaperones for its role in folding actin and other proteins, and the interaction of various co-chaperones with CCT determines the outcome of the final folding process. A previous study has confirmed that ARV p17 regulates PTEN activity via CK2 in the cytoplasm (48). The CK2 is involved in various cellular functions, including cell division, growth, and gene expression (57, 58). Additionally, CK2 promotes Hsp90 activity through the phosphorylation of Cdc37 (59, 60). The finding reveals that ARV p17 stimulates the phosphorylation of Cdc37 at Ser13, which, in turn, facilitates its binding with Hsp90 and reinforces Hsp90 activity. However, when we used the inhibitor TBB to suppress CK2, ARV p17 was unable to effectively phosphorylate Cdc37 at Ser13, demonstrating that ARV p17 activates the Hsp90-Cdc37 complex via CK2 (51). Conversely, the P protein of the rabies virus requires assistance from Hsp90 but is not dependent on the formation of the Hsp90-Cdc37 complex. Even without Cdc37 phosphorylation at Ser13, Hsp90 is still able to support the expression and stability of the P protein of the rabies virus (61).

TRiC plays a crucial role in MRV replication by assisting the folding of the viral σ3 outer-capsid protein, which is essential for its assembly into viral particles (39). Although we demonstrated that the p17 protein of ARV modulates molecular chaperone TRiC to protect outer-capsid protein σC and inner core protein σA and non-structural protein σNS from ubiquitin-proteasome degradation (50), it is still unclear which co-chaperone interacts with TRiC to stabilize viral proteins. This study further demonstrated that the p17 protein of ARV enhances the interaction between PhLP1 and CCT2 via CK2-mediated phosphorylation of PhLP1 (52), suggesting that p17 plays an important role in modulating the formation of the TRiC-PhLP1 complex (53, 54), which chaperones the σC and σA proteins of ARV (50). This is the first report to reveal a regulatory role for PhLP1 in CCT2-dependent folding or stabilization of viral proteins. The current and past studies reveal that Hsp90-Cdc37 and TRiC-PhLP1 chaperone complexes are essential for chaperoning p17, σC, σA, and σNS proteins of ARV, respectively. The binding of PhLP1 to CCT2 diminishes Hsc70’s association with TRiC, leading to IκBα degradation, thereby subsequent activation of NF-κB. This study suggests that PhLP1 and Hsc70 exhibit a competitive relationship in binding to TRiC, with ARV stimulation causing a shift in their balance. The current study provides the first evidence to reveal that the TRiC-Hsc70 chaperone machinery plays a critical role in protecting IκBα from ubiquitin-proteasome-mediated degradation.

Previous studies have confirmed that Hsp90 enhances the activation of the transcription factor NF-κB (62, 63). In the early stages of ARV infection, NF-κB activation is enhanced to delay the onset of apoptosis (64). The present study demonstrates that the p17 protein of ARV indeed activates the IKK-NF-κB signaling through the CK2/Hsp90/Cdc37 pathway, which in turn induces chaperone-mediated autophagy. Hsp90 activates NF-κB by enhancing the stability of the IKK protein, which leads to phosphorylation and ubiquitination of IκBα, ultimately resulting in its degradation (65). Previous studies have suggested that several viruses, such as HIV, human T-lymphotropic virus 1 (HTLV-1), hepatitis B virus, and influenza virus, can trigger NF-κB activation. The activated NF-κB supports viral replication, postpones virus-induced cell death, or aids in immune response mediation (8, 66). In some dsRNA viruses, NF-κB is activated via the accumulation of viral RNA, which subsequently activates the dsRNA-dependent kinase PKR (67, 68). An earlier study has demonstrated that the X protein of HBV regulates cellular signaling pathways by activating NF-κB through Ras and MEKK-1 (69). HTLV-1’s Tax protein has been shown to bind directly with IKK (70, 71), which promotes NF-κB activation, leading to enhanced cellular gene expression that promotes cell growth and transforms cells into malignant tumor cells (8, 72). This study demonstrates that ARV p17 transcriptionally upregulates Beclin1 via the NF-κB signaling, enhancing cellular autophagy. Autophagy, which serves as a cellular defense mechanism, is manipulated by viruses to enhance their replication. When Sindbis virus infects the mouse brain, artificially increasing Beclin1 expression significantly reduces viral titers (73). The VP1 protein of foot-and-mouth disease virus binds to SQSTM1/p62 and promotes autophagosome formation, facilitating viral entry and replication within host cells (74). Hepatitis C virus stimulates the formation of autophagosomes but blocks their maturation, leading to autophagosome accumulation and creating a favorable environment for viral replication in the cell (75). Many viruses have been reported to regulate autophagy to facilitate their replication. The current and past studies from our laboratory have demonstrated that ARV p17 induces autophagy to enhance viral replication (43, 47). The mechanisms underlying p17-induced chaperone-mediated autophagy benefiting viral replication remain unclear and warrant future investigations to elucidate its mechanism.

**Fig 10:**
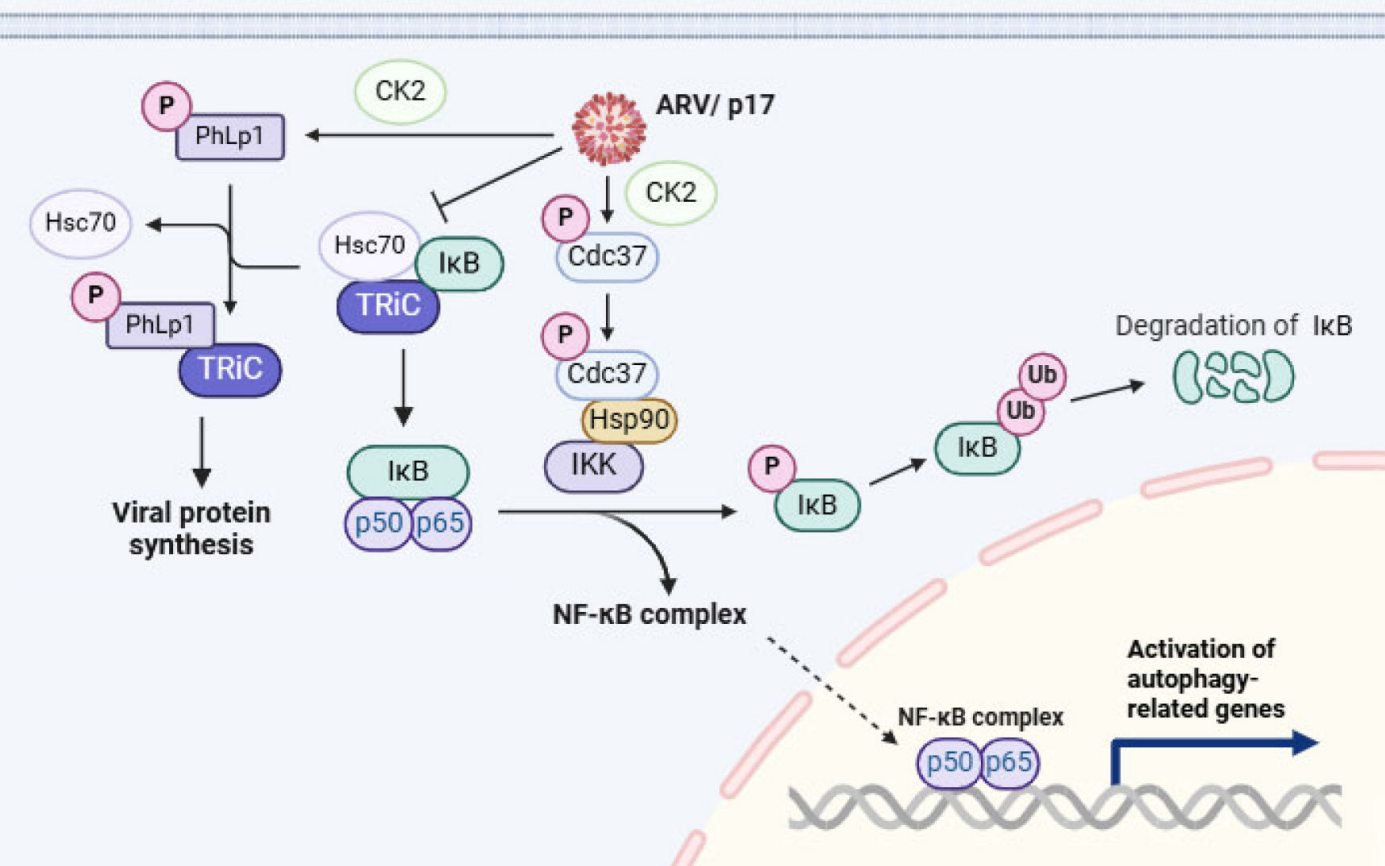
ARV modulates the IKK/NF-κB pathway via the TRiC and Hsp90 chaperones, promoting chaperone-mediated autophagy. A model is proposed to illustrate that ARV p17 regulates the IKK/NF-κB pathway by modulating TRiC/co-chaperone interactions and CK2/Hsp90/Cdc37 signaling, thereby activating chaperone-mediated autophagy to enhance viral replication. Our previous study suggested that p17 mediates phosphorylation of Cdc37 through CK2 (51). Phosphorylation of PhLP1 is mediated by the protein kinase CK2 (52). The figure was created with BioRender.com.

A model is proposed to illustrate that ARV p17 modulates the suppression of IκB by regulating co-chaperone PhLP1 and Hsc70 binding to TRiC, and activation of the CK2-Hsp90-Cdc37-IKK-NF-κB pathway to induce chaperone-mediated autophagy (Fig. 10). This study sheds additional light on how ARV modulates the cellular autophagy pathway, which may also provide a novel perspective for developing antiviral approaches.

## MATERIALS AND METHODS

### Cells and viruses

The S1133 strain of ARV was used in this study. Vero lineage was isolated from an African green monkey, while the A549 cancer cell line was from adenocarcinomic human alveolar basal epithelial cells. Both cell lines were grown in minimum essential medium or HAM’s F-12K (ThermoFisher Scientific, MA, USA) medium supplemented with 10% fetal bovine serum, 10 mM HEPES (pH 7.2; ThermoFisher Scientific, MA, USA), and 1% Penicillin/Streptomycin (ThermoFisher Scientific, MA, USA) at 37°C in a 5% CO_2_ humidified incubator. Once the cells reached a monolayer, they were prepared for passaging under sterile conditions in a laminar flow hood. The cells were washed twice with 1× PBS and then digested using trypsin-EDTA. After cells were resuspended, fresh culture medium containing FBS was added to neutralize the trypsin activity. The cells were evenly transferred into a 4-well plate for subsequent experiments.

### Reagents and antibodies

HSF1A (TRiC inhibitor), TBB (CK2 inhibitor), VER-155008 (Hsc70 inhibitor), and Bay11-7085 (NF-κB inhibitor) were purchased from MedChemExpress (Monmouth Junction, USA). Monoclonal antibodies against p17, σA, σC, and σNS proteins of ARV were prepared in our laboratory as described previously (46, 76, 77). The primary and secondary antibodies used in this study, along with their catalog numbers and working dilution, are detailed in Table 1.

**TABLE 1:** The catalog numbers and dilution factors of the respective antibodies used in this study

Antibodies	Catalog numbers	Clone name	Dilution factors	Manufacturer
^*a*^Mouse anti-p17	–^*b*^	–	2,000	Our laboratory (44)
Mouse anti-σA	–	–	4,000	Our laboratory (76)
Mouse anti-σC	–	–	4,000	Our laboratory (77)
Mouse anti-casein kinase IIα	sc-12738	1AD9	1,000	Santa Cruz Biotechnology
Rabbit anti-CK2 substrate ([pS/pT]DXE)	8738	–	1,000	Cell Signaling Technology
Mouse anti-IKKα	11930	3G12	2,000	Cell Signaling Technology
Rabbit anti-p-IĸBα (S32)	2859	14D4	1,000	Cell Signaling Technology
Mouse anti-IĸBα	4814	L35A5	2,000	Cell Signaling Technology
Rabbit anti-NF-κB p65	4764	C22B4	2,000	Cell Signaling Technology
Rabbit anti-NF-κB p50	3035	–	1,000	Cell Signaling Technology
Mouse anti-PhLP	sc-376918	A-8	1,000	Santa Cruz Biotechnology
Rabbit anti-PFDN5	ab129116	FPR7755	2,000	Abcam
Rabbit anti-CCT2	ab92746	EPR4084	3,000	Abcam
Rabbit anti-CCT5	ab129016	EPR7562	3,000	Abcam
Rabbit anti-HSPA8	8444	D12F2	2,000	Cell Signaling Technology
Rabbit anti-Hsp90	4877	C45G5	2,000	Cell Signaling Technology
Rabbit anti-p-Cdc37 (S13)	13248	D8P8F	2,000	Cell Signaling Technology
Mouse anti-Cdc37	sc-17758	C-11	2,000	Santa Cruz Biotechnology
Mouse anti-ubiquitin	sc-8017	P4D1	1,000	Santa Cruz Biotechnology
Rabbit anti-LAMP2	49067	D5C2P	2,000	Cell Signaling Technology
Rabbit anti-Beclin1	3495	D40C5	2,000	Cell Signaling Technology
Rabbit anti-LC3B	3868	D11	2,000	Cell Signaling Technology
Rabbit anti-PI3 Kinase Class III	4263	D9A5	2,000	Cell Signaling Technology
Rabbit anti-β-tubulin	2128	9F3	2,000	Cell Signaling Technology
Mouse anti-GAPDH	ab8245	6C5	3,000	Abcam
Rabbit anti-Histon H2A	2578	–	2,000	Cell Signaling Technology
Mouse anti-β-actin	MAB1501	C4	10,000	Merck Millipore
Goat anti-mouse IgG (H + L) HRP	5220-0341	–	5,000	SeraCare
Goat anti-rabbit IgG (H + L) HRP	5220-0336	–	5,000	SeraCare

^*a*^ Polyclonal antibodies.

^*b*^ “–” indicates not applicable.

### shRNAs used in this study

The pLKO-AS1-puro plasmid encoding shRNAs was obtained from the National RNAi Core Facility, Academia Sinica, Taiwan. The target sequences for casein kinase II (TRCN0000000607) and Hsp90 (TRCN0000315007) were 5-CGTAAACAACACAGACTTCAA-3 and 5-CGTAAACAACACAGACTTCAA-3, respectively. The target sequence for Hsc70 (TRCN0000221653) was 5-GCTCGATTT GAGGAGTTGAAT-3. The shRNA with the most significant downregulating effect for a respective gene was selected and used in this study. The cells were transfected with the respective shRNA for 6 h, followed by infection with ARV at an MOI of 10 for 24 h, respectively. Whole cell lysates were collected for Western blot analysis.

### Reverse transcription and polymerase chain reaction, plasmid construction, and transient transfection

The pCI-Flag-p17 constructs were generated as demonstrated previously (44). For the construction of the pCI-Flag-CK2 plasmid, gene fragments were amplified by PCR using specific primers shown in Table 2. The total RNA was extracted from ARV-infected Vero cells using TRIzol solution (ThermoFisher Scientific, MA, USA) according to the manufacturer’s protocol. A total of 3 µL of total RNA was used as templates for reverse transcription (RT) with M-MLV reverse transcriptase (Promega Co., Madison, USA) using the respective primers (Table 2). Reverse transcription was carried out at 42°C for 15 min and 72°C for 15 min. PCR was performed with 1 µL of cDNA, 1 µL of each primer, 2 µL of PCR mix, and 15 µL of ddH_2_O, in a total volume of 20 µL. The PCR conditions for amplification were 95°C for 5 min, 35 cycles of 95°C for 30 s, 55°C for 30s, and extension at 72°C for 30 s (p17) or 2 min (CK2), followed by 72°C for 10 min for a final extension. The PCR products were electrophoresed in a 1% agarose gel and were then purified using an agarose gel DNA extraction kit (Bio kit, Taiwan). Purified PCR products were digested with the respective enzymes, followed by ligation into the same restriction sites of the pCI-neo-Flag plasmid to generate pCI-Flag-p17 and CK2 plasmids, respectively. All constructs were confirmed by DNA sequencing.

**TABLE 2:** The primers used in this study

Gene	Accession number	Sequence (5′−3′)	Location	Expected size (bp)
Generation of pCI-Flag-p17 and Ck2 plasmids for transfection				
p17 (full)	AF330703	F: CGGAATTCATGCAATGGCTCCGCCATACGA (EcoR I) R: GCTCTAGATCATAGATCGGCGTCAAATCGC (Xba I)	293–314 733–712	441
Casein kinase 2	KF741763.1	F: TCTAGAACGATGGCGCGTGCCATATACGACTT (Xba I) R: GTCGACCCTAGGCGGTAAAAGTGGC (Sal I)	16–38 1,266–1,249	1,251
Primers for semi-quantitative RT-PCR				
Beclin1	NM_001266685	F: CATTACTTACCACAGCCCAG R: CTGAGTGTCCAGCTGGTCTA	134–154 454–433	321
GAPDH	NM_001266685	F: CACCACCATGGAGAAGGCTGGGGCTCA R: GGCAGGTTTCTCCAGACGGCAGGTCAG	480–506 933–907	454

For transfection, cells were grown to 70%–80%, washed twice with serum-free medium, and supplemented with fresh FBS-free medium. Plasmid DNA was mixed with the transfection reagent (SignaGen Laboratories, MD, USA) following the manufacturer’s recommended protocol. The prepared mixture was then added to the culture dish. After 6 h of transfection, the supernatant with the transfection reagent was removed. The cells were washed twice with serum-free medium, followed by the addition of fresh FBS-containing medium. Cells were maintained in a 37°C incubator with 5% CO_₂_, and samples were collected at the indicated time points for further analysis.

### Proximity ligation assays

The Duolink commercial assay (Sigma-Aldrich, St. Louis, USA) was used to detect the LC3-CCT2 interaction in ARV-infected Vero cells. PLA enables the detection of direct protein-protein interactions within a range of approximately 40 nm in fixed, intact cells (78). Vero cells were cultured on 18 × 18 mm coverslips, and after 24 h of ARV infection, they were fixed with 4% paraformaldehyde (ThermoFisher Scientific, MA, USA) for 10 min at room temperature. Cells were treated with a blocking buffer for 30 min at 37°C, followed by incubation with specific primary antibodies. Following several washing steps, PLA probes linked to anti-mouse and anti-rabbit secondary antibodies were applied to the cells. Next, the cells underwent ligation and amplification reactions. Following two washes with 1× PBS, the cells were stained with 4′,6-diamidino-2-phenylindole (DAPI; Cell Signaling, St. Louis, USA) for 10 min. The cells were mounted on glass slides using ibidi mounting medium (Ibidi GmbH, Gräfelfing, Germany) and examined using a BX51 fluorescence microscope.

### Cytoplasmic and nuclear protein isolation

The transfected cells were harvested using trypsin-EDTA and transferred to microcentrifuge tubes. The samples were centrifuged at 800 × *g* for 10 min at 4°C. After discarding the supernatant, the cells were resuspended in 1× PBS and washed twice using the same step. Cytoplasmic and nuclear protein extraction was carried out using the Compartmental Protein Extraction Kit (BioChain, CA, USA). The washed cells were resuspended in 200 µL of buffer C and agitated at 4°C for 20 min. Membrane disruption was achieved by repeatedly drawing the samples (~100 times) through a 26.5-gauge syringe. The lysates were then centrifuged at 15,000 × *g* for 20 min at 4°C, and the supernatant was collected as the cytoplasmic protein fraction. The pellet was resuspended in 400 µL of buffer W, mixed thoroughly, and shaken at 4°C for 5 min. The mixture was then centrifuged at 15,000 × *g* for 20 min at 4°C, and the supernatant was discarded. The pellet was resuspended in 100 µL of buffer N and shaken at 4°C for 20 min. The mixture was then centrifuged at 15,000 × *g* for 20 min at 4°C, and the resulting supernatant was collected as the nuclear protein fraction.

### Co-immunoprecipitation assays

The Catch and Release version 2.0 kit was used in this study (Merck Millipore, MA, USA). The cells were washed twice with 1× PBS and lysed in 200 µL of CHAPS lysis buffer (40 mM HEPES [pH 7.5], 1 mM EDTA, 10 mM glycerophosphate, 120 mM NaCl, 50 mM NaF, 10 mM pyrophosphate, and 0.3% CHAPS). One thousand micrograms of total proteins collected from each sample was incubated with 2 µg of the indicated antibodies or rabbit IgG (negative control). Sample proteins, antibodies, and resin were sequentially incubated together and incubated at 4°C for 12 h following the manufacturer’s instructions. After removing the supernatant, 400 µL of 1× wash buffer was added to the column. The column was centrifuged at 4°C, 2,000 × *g* for 30 s, the filtrate was discarded, and the washing step was repeated three times. Finally, 70 µL of elution buffer was added, mixed gently, and incubated on ice for 5 min. The column was centrifuged at 4°C, 2,000 × *g* for 30 s, and the supernatant was collected as the sample for analysis. The immunoprecipitated proteins were separated by SDS-PAGE followed by Western blot analysis with the indicated antibodies.

### Electrophoresis and Western blot assays

Cells were cultured in 4-well plates 24 h before performing the virus infection or plasmid transfection. Collected cells were washed twice with 1× PBS and lysed with lysis buffer (Cell Signaling, St. Louis, USA). The protein concentrations in the cell lysates were measured using the Bio-Rad Protein Assay (Bio-Rad Laboratories, CA, USA) according to the manufacturer’s protocol. An equal quantity of each sample was mixed with 2.5× Laemmli loading buffer (Bio-Rad Laboratories, CA, USA) and incubated in boiling water for 10 min. Following electrophoresis on an SDS-polyacrylamide gel, the samples were transferred to a PVDF membrane (GE Healthcare Life Sciences, Chicago, USA). The detection of protein expression was carried out using specific primary antibodies along with HRP-labeled secondary antibodies. X-ray films (Kodak, Rochester, USA) were used to capture the results after the membrane was exposed to ECL Plus reagent (Cytiva, MA, USA). Quantification of target proteins was performed using ImageJ.

### Detection of LC3 puncta

To clarify the effects of ARV infection on autophagy, cells were transfected with the mCherry-LC3 or GFP-LC3 plasmids to observe LC3 puncta fluorescence following ARV infection. For the experiment, Vero cells were seeded on 18 × 18 mm coverslips. Following treatment, the cells were washed twice with 1× PBS and then fixed at the indicated times with 4% paraformaldehyde (Alfa Aesar, MA, USA) for 20 min at room temperature. Subsequently, the fixed cells were incubated in PBS containing 0.1% Triton X-100 (Sigma-Aldrich, T9284) for 10 min. After washing twice with 1× PBS, the cells were blocked with SuperBlock Blocking Buffer for 1 h at room temperature. The cells were washed twice with 1× PBS and stained with DAPI for 10 min. After washing the cells three times with 1× PBS, they were mounted onto glass slides using ibidi mounting medium, followed by observation with a BX51 fluorescence microscope.

### Statistical analysis

All data obtained in this study were evaluated for Duncan’s multiple range test using Prism 8 software (GraphPad, San Diego, CA, USA) and the Student’s *t*-test. The data were expressed as averages of three independent experiments. *P* values < 0.05 were considered statistically significant.

## Data Availability

The data described in the article are available in the figures or supplemental tables.

## SUPPLEMENTAL MATERIAL

Supplemental figures jvi.01089-25-s0001.pdf Figures S1 to S7.

ASM does not own the copyrights to Supplemental Material that may be linked to, or accessed through, an article. The authors have granted ASM a non-exclusive, world-wide license to publish the Supplemental Material files. Please contact the corresponding author directly for reuse.
