# Supplementary material for: The oncolytic avian reovirus p17 protein triggers chaperone-mediated autophagy by modulating Hsp90 and the T-complex protein-1 ring complex chaperones and co-chaperones to activate the IKK/NF-κB signaling
Source: J Virol. 2025 Dec 3;99(12):e01089-25. doi: 10.1128/jvi.01089-25 (PMC12724251; doi:10.1128/jvi.01089-25)

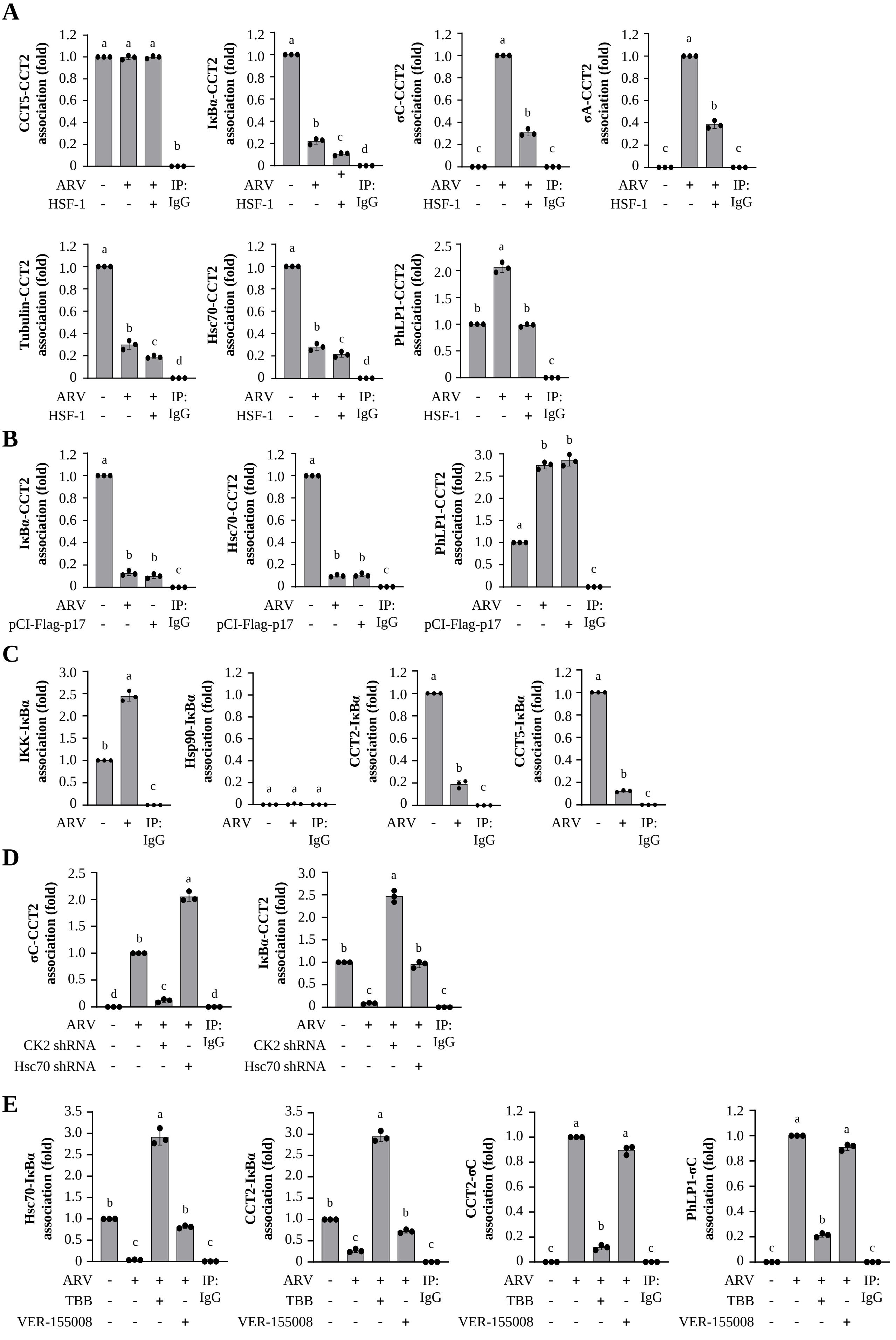
A
CCT5-CCT2 association (fold)
IκBα-CCT2 association (fold)
σC-CCT2 association (fold)
σA-CCT2 association (fold)
Tubulin-CCT2 association (fold)
Hsc70-CCT2 association (fold)
PhLP1-CCT2 association (fold)
ARV
HSF-1
IP: IgG
B
IκBα-CCT2 association (fold)
Hsc70-CCT2 association (fold)
PhLP1-CCT2 association (fold)
ARV
pCI-Flag-p17
IP: IgG
C
IKK-IκBα association (fold)
Hsp90-IκBα association (fold)
CCT2-IκBα association (fold)
CCT5-IκBα association (fold)
ARV
IP: IgG
D
σC-CCT2 association (fold)
IκBα-CCT2 association (fold)
ARV
CK2 shRNA
Hsc70 shRNA
IP: IgG
E
Hsc70-IκBα association (fold)
CCT2-IκBα association (fold)
CCT2-σC association (fold)
PhLP1-σC association (fold)
ARV
TBB
VER-155008
IP: IgG

F

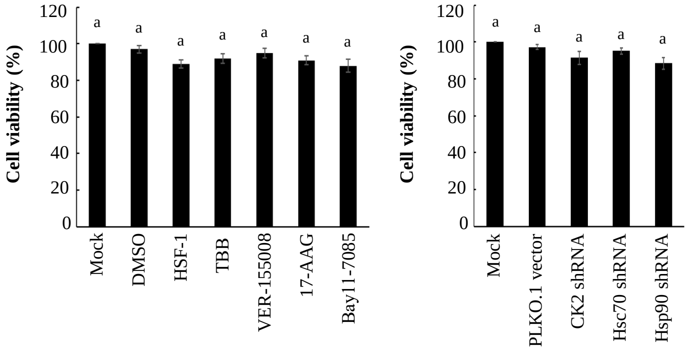

G

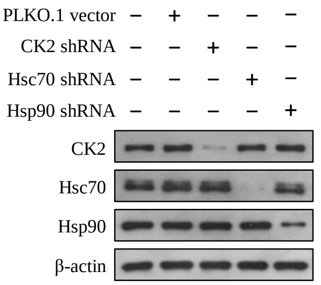

Figure S1. ARV modulates TRiC chaperone complexes to regulate IκB protein expression. Immunoblots from Figure 1 panels A-E were quantitated by densitometric analysis using ImageJ software. The levels of the indicated proteins in the mock treatment were considered 1-fold. The Duncan's Multiple Range Test (DMRT) was used to analyze the statistical significance of all data obtained using SPSS software (Version 20.0). The image shown is from a single experiment that is representative of at least three separate experiments. (F) To investigate whether inhibitors or shRNAs used in this study have deleterious effects on the cell, the viability of the cells was assessed by an MTT assay. Each value represents three independent experiments. (G) To validate the effectiveness of the shRNAs used in this study, we evaluated their knockdown efficiency by Western blot analysis.

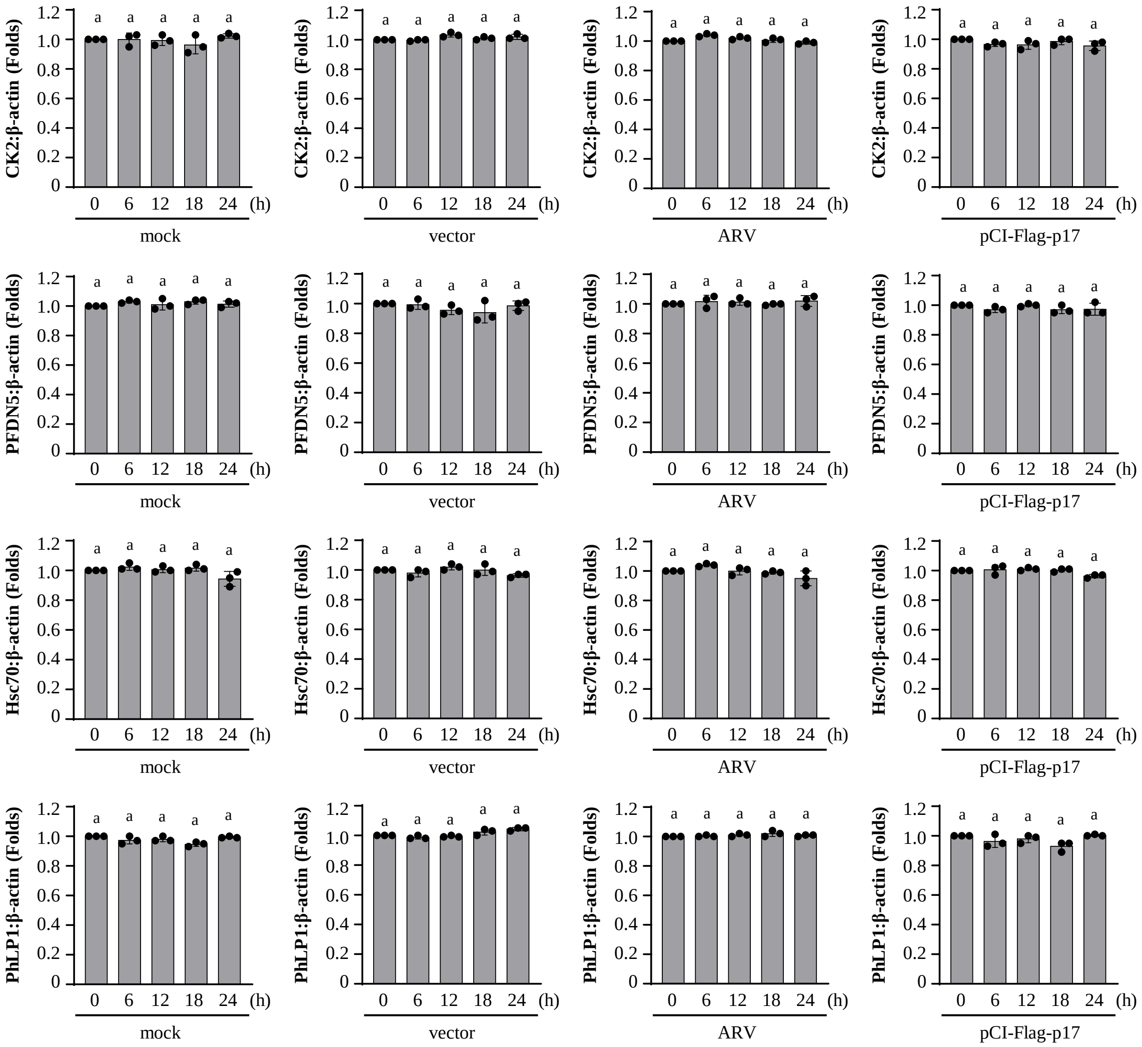

Figure S2. ARV did not modulate TRiC co-chaperone proteins expression. Immunoblots from Figure 2 were quantitated by densitometric analysis using ImageJ software. The levels of the indicated proteins in the mock treatment were considered 1-fold. The Duncan's Multiple Range Test (DMRT) was used to analyze the statistical significance of all data obtained using SPSS software (Version 20.0). The image shown is from a single experiment that is representative of at least three separate experiments.

Cytoplasmic extract

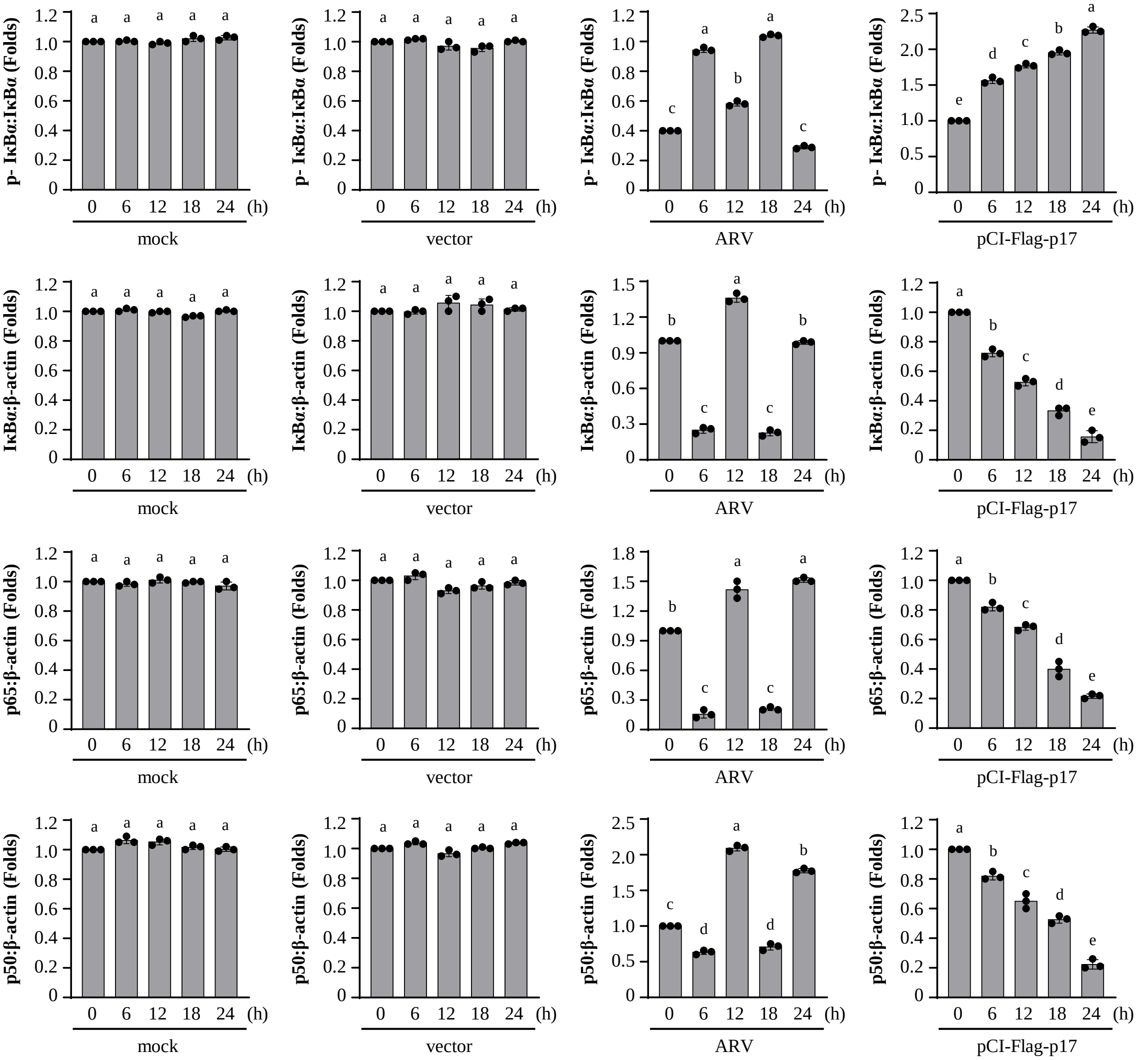

Nuclear extract

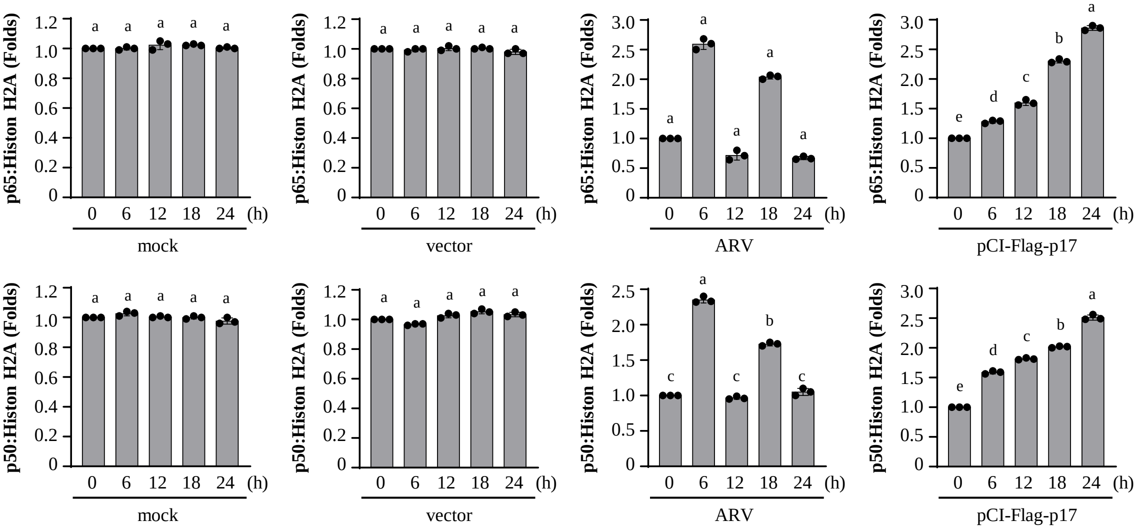

Figure S3. ARV p17 activated NF-κB pathway. At the designated time points, Vero cells were infected with ARV and transfected with pCI-Flag-p17. Immunoblots from Figure 4 were quantitated by densitometric analysis using ImageJ software. The levels of the indicated proteins in the mock treatment were considered 1-fold. The Duncan's Multiple Range Test (DMRT) was used to analyze the statistical significance of all data obtained using SPSS software (Version 20.0). The image shown is from a single experiment that is representative of at least three separate experiments.

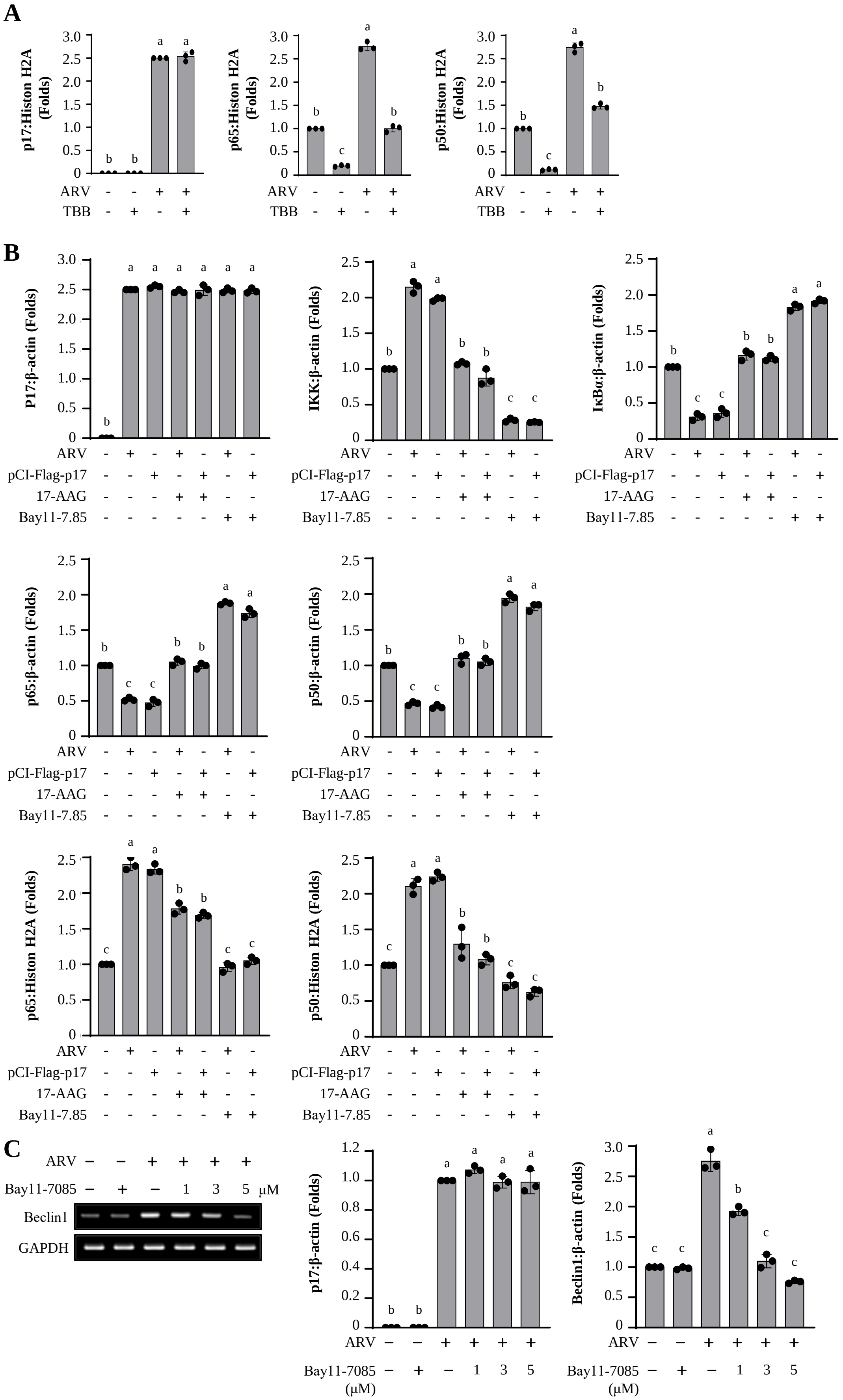
A
p17:Histon H2A (Folds)
p65:Histon H2A (Folds)
p50:Histon H2A (Folds)
ARV
TBB
B
P17:β-actin (Folds)
IKK:β-actin (Folds)
IκBα:β-actin (Folds)
p65:β-actin (Folds)
p50:β-actin (Folds)
p65:Histon H2A (Folds)
p50:Histon H2A (Folds)
ARV
pCI-Flag-p17
17-AAG
Bay11-7.85
C
ARV
Bay11-7085
μM
Beclin1
GAPDH
p17:β-actin (Folds)
Beclin1:β-actin (Folds)
Bay11-7085 (μM)

Figure S4. ARV p17 stimulates autophagy by activating the Hsp90/NF-kB signaling pathway. Immunoblots from Figure 6 panels (A)(B)(D) were quantitated by densitometric analysis using ImageJ software. The levels of the indicated proteins in the mock treatment were considered 1-fold. The Duncan's Multiple Range Test (DMRT) was used to analyze the statistical significance of all data obtained using SPSS software (Version 20.0). The image shown is from a single experiment that is representative of at least three separate experiments. (C) To examine the mRNA levels of Beclin1, semi-quantitative RT-PCR was performed on ARV-infected and mock-infected Vero cells in the presence or absence of different concentrations of Bay11-7085. The PCR products were separated by electrophoresis on an agarose gel and stained with ethidium bromide.

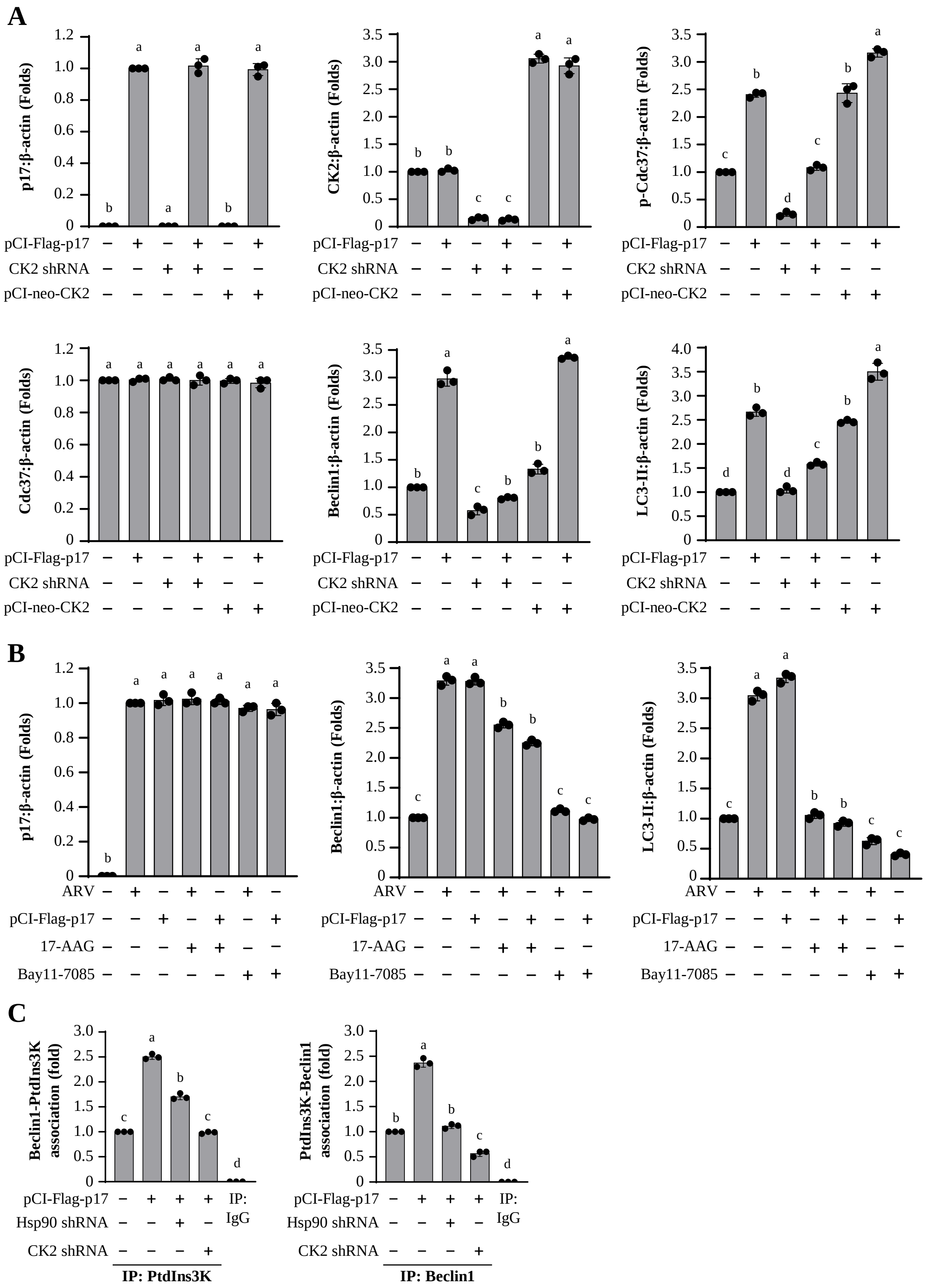

Figure S5. ARV p17 enhances the PtdIns3K-Beclin1 complex, triggering autophagosome formation. Immunoblots from Figure 7 panels A-C were quantitated by densitometric analysis using ImageJ software. The levels of the indicated proteins in the mock treatment were considered 1-fold. The Duncan's Multiple Range Test (DMRT) was used to analyze the statistical significance of all data obtained using SPSS software (Version 20.0). The image shown is from a single experiment that is representative of at least three separate experiments.

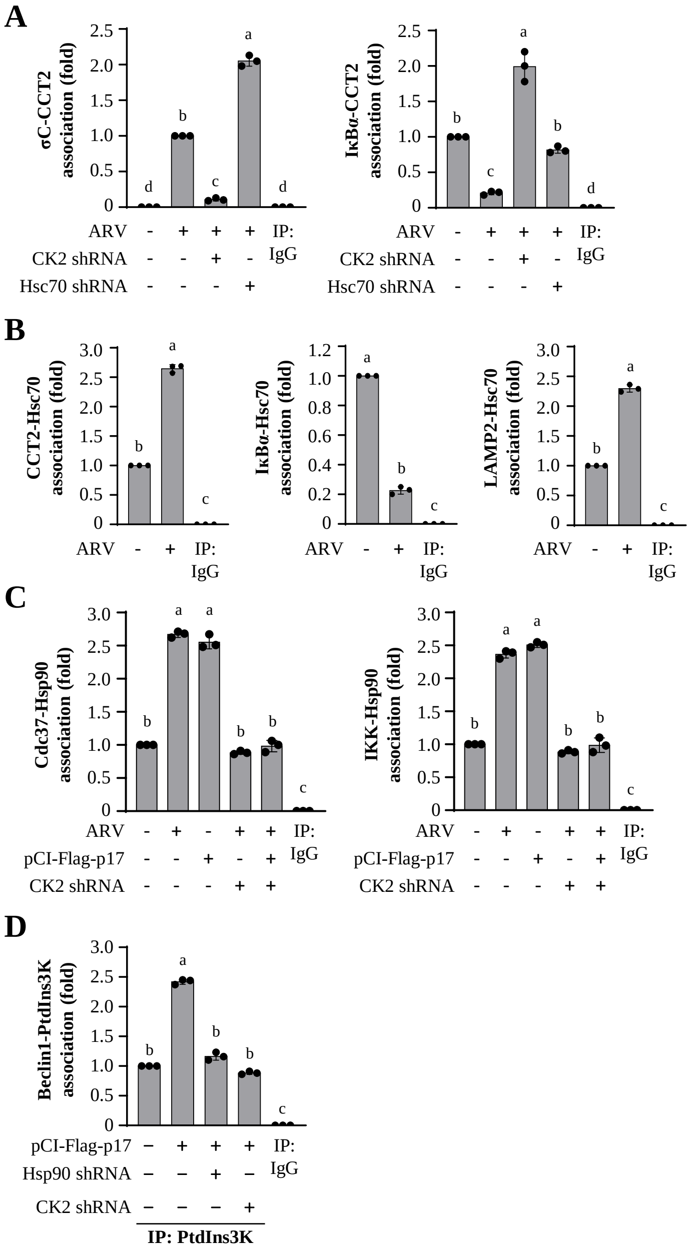

Figure S6. ARV p17 promotes autophagy via the Hsp90/NF-κB signaling pathway in A549 cancer cells. Immunoblots from Figure 9 panels A-D were quantitated by densitometric analysis using ImageJ software. The levels of the indicated proteins in the mock treatment were considered 1-fold. The Duncan's Multiple Range Test (DMRT) was used to analyze the statistical significance of all data obtained using SPSS software (Version 20.0). The image shown is from a single experiment that is representative of at least three separate experiments.

Figure S7. Original blots and images. All original blots and images of Fig. 1-9 are shown.

Figure 1A

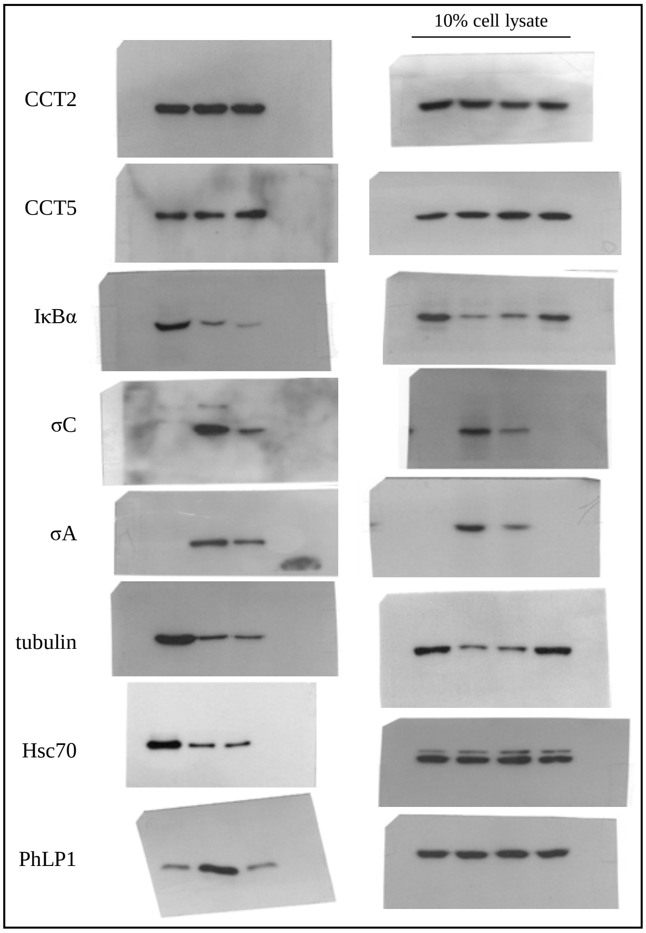

Figure 1B

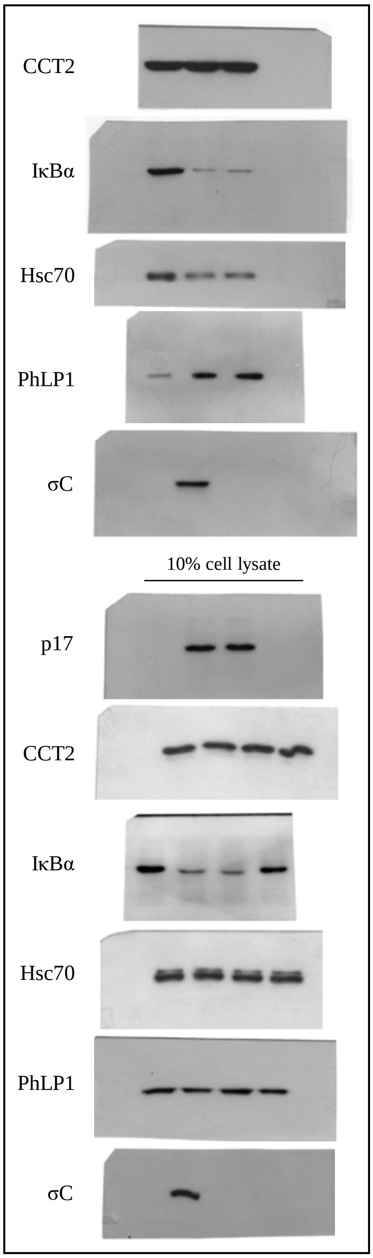

Figure 1C

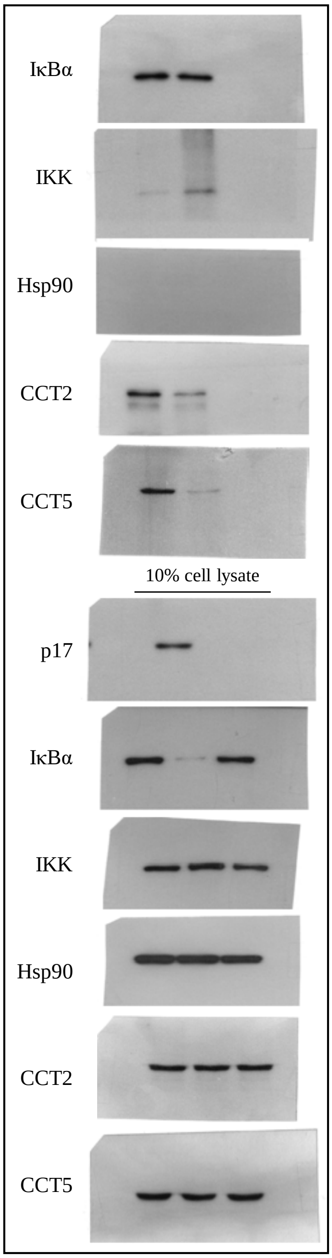

Figure 1D

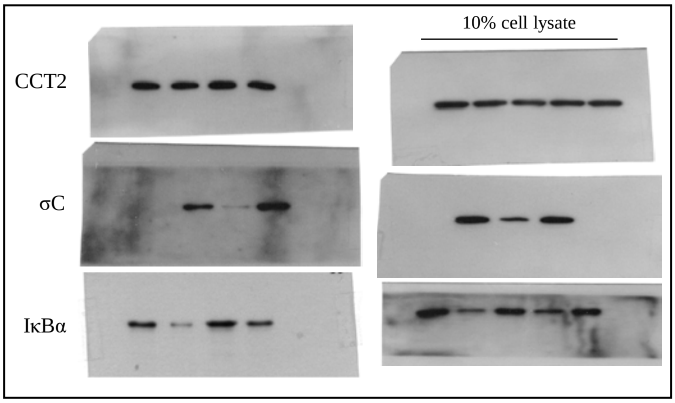

Figure 1E

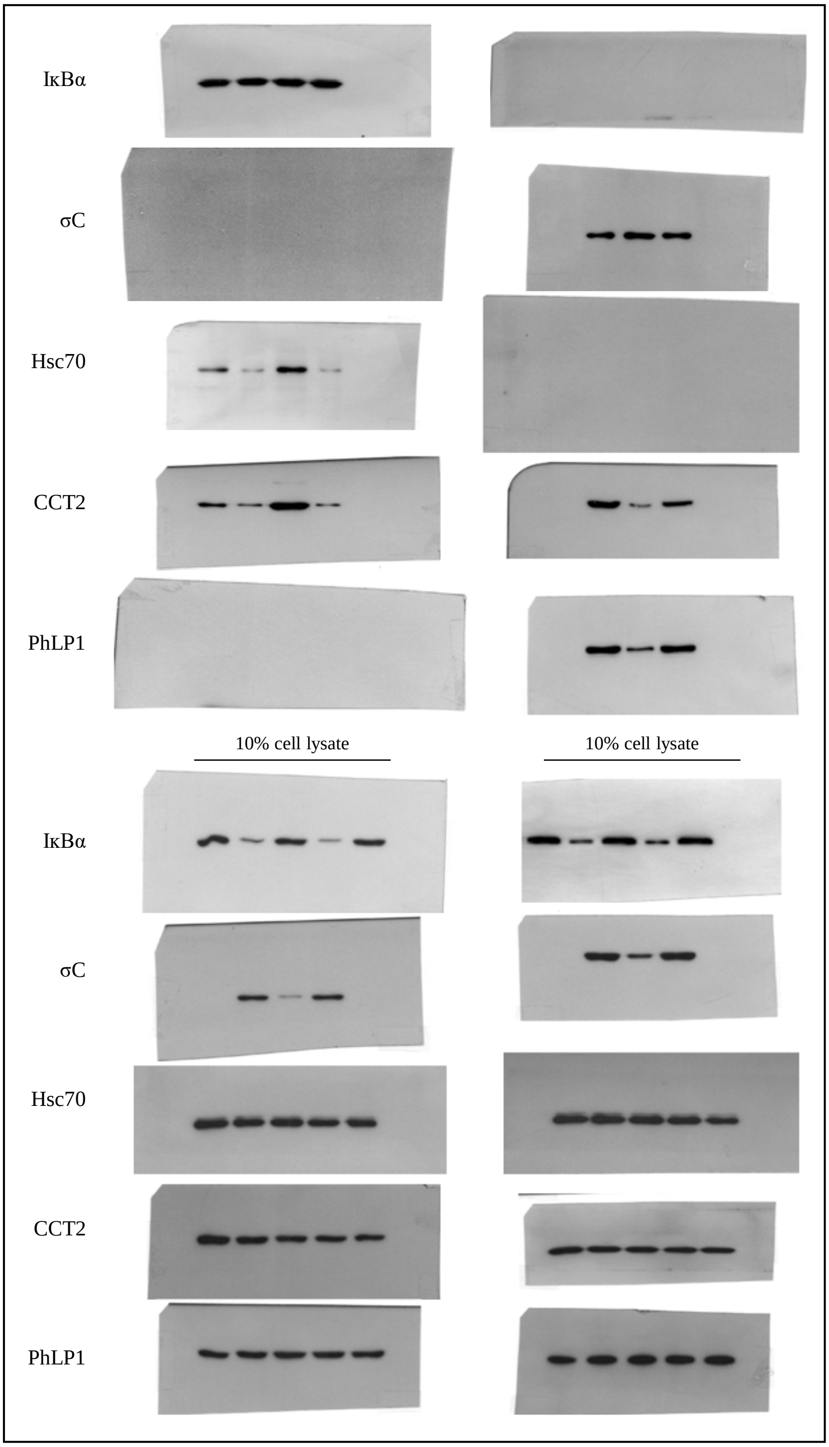

IκBα
σC
Hsc70
CCT2
PhLP1
10% cell lysate
10% cell lysate
IκBα
σC
Hsc70
CCT2
PhLP1

Figure 2A

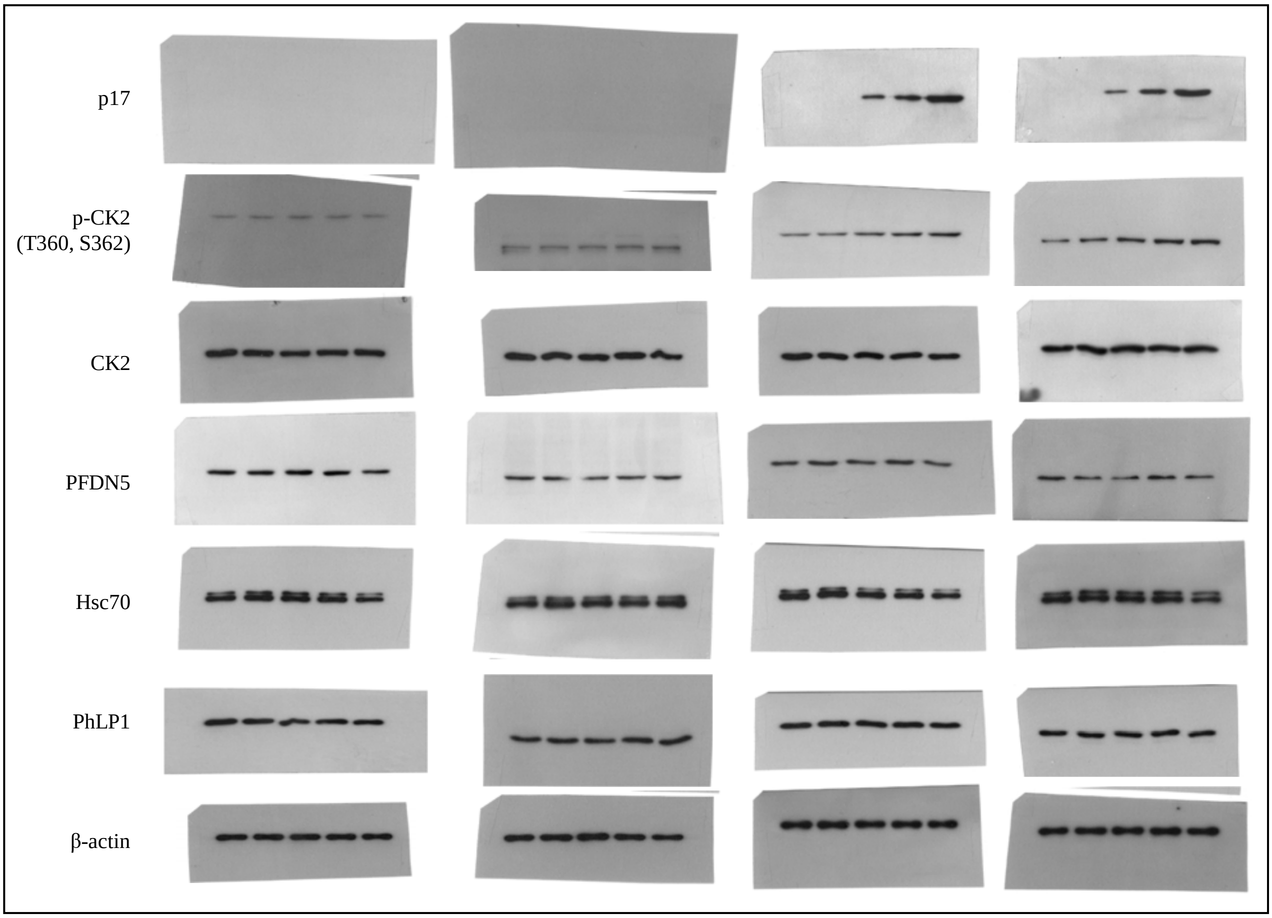

Figure 3A

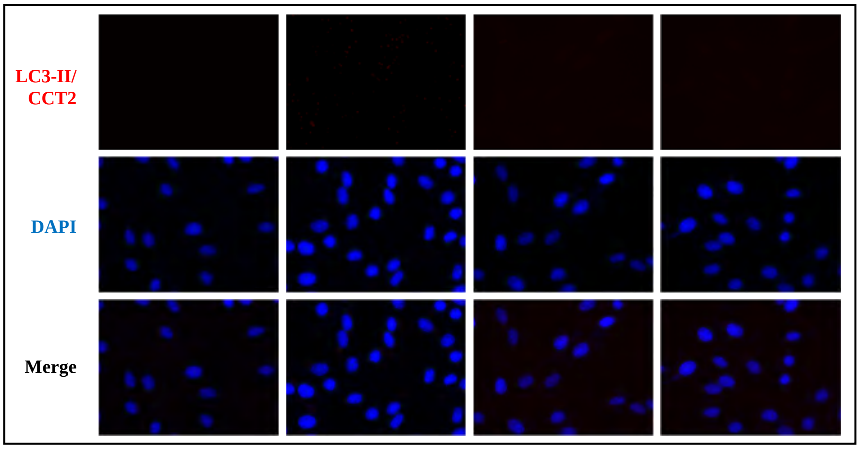

Figure 3C

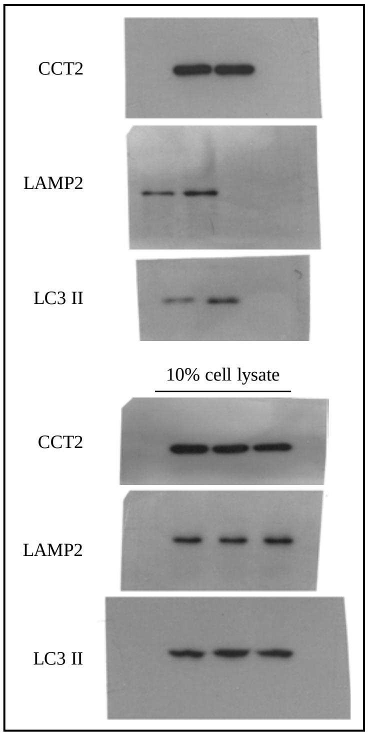

Figure 3D

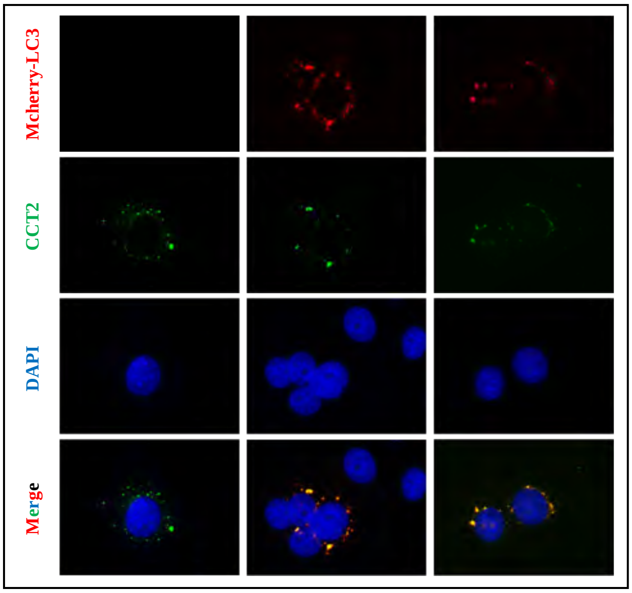

Figure 4

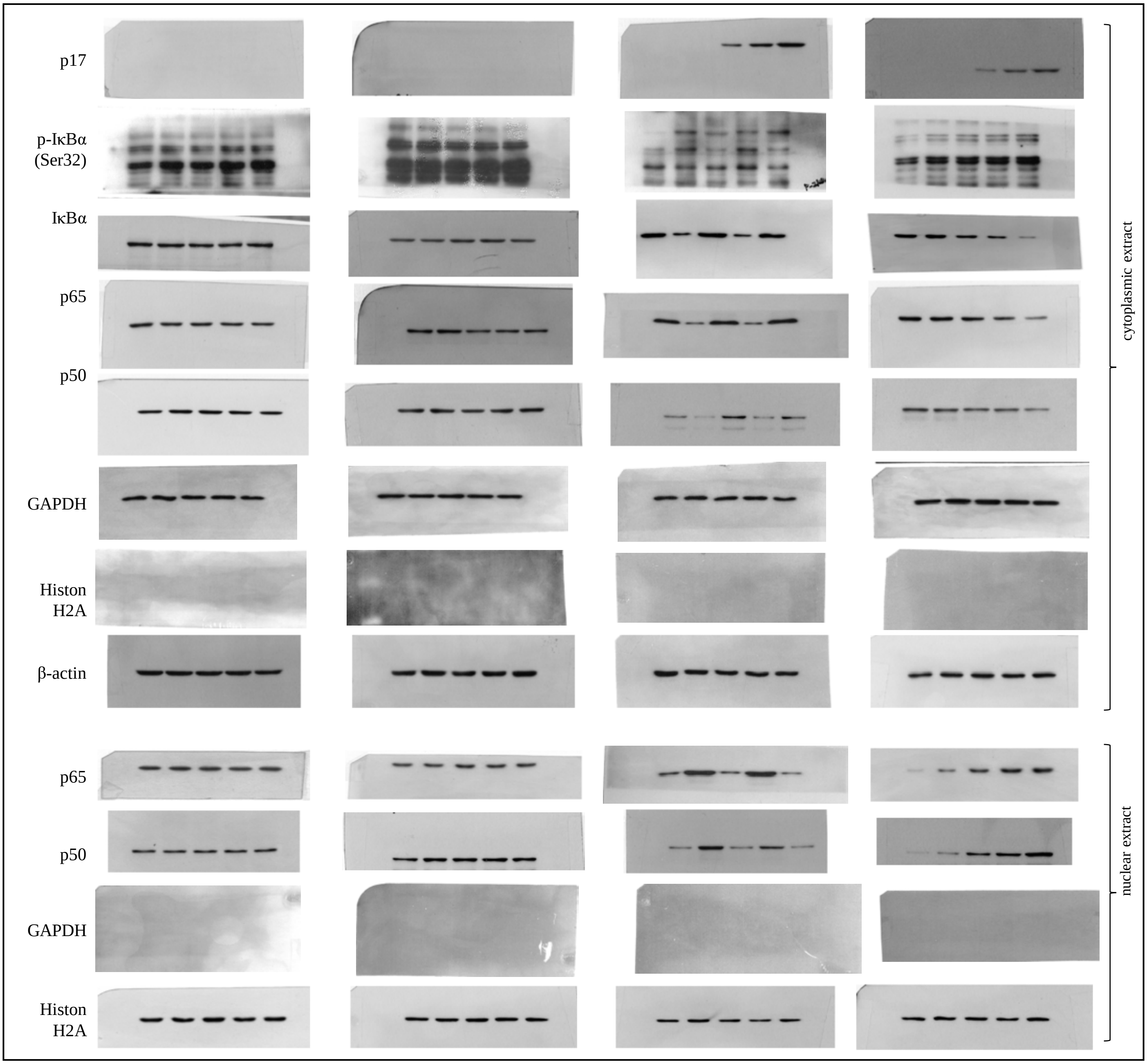

Figure 5A

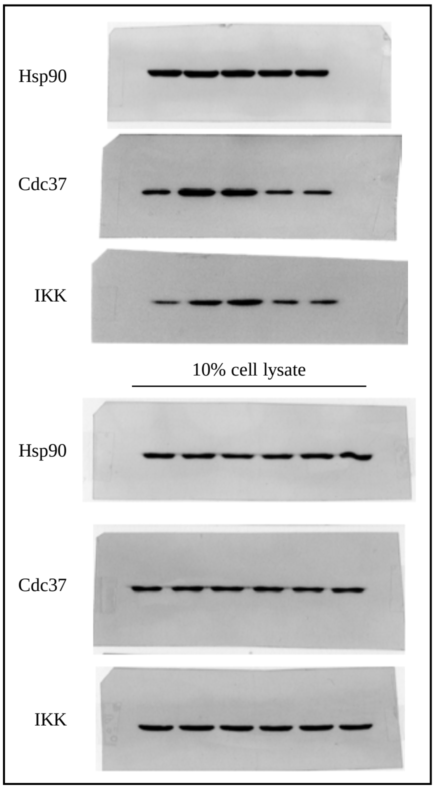

Figure 5C

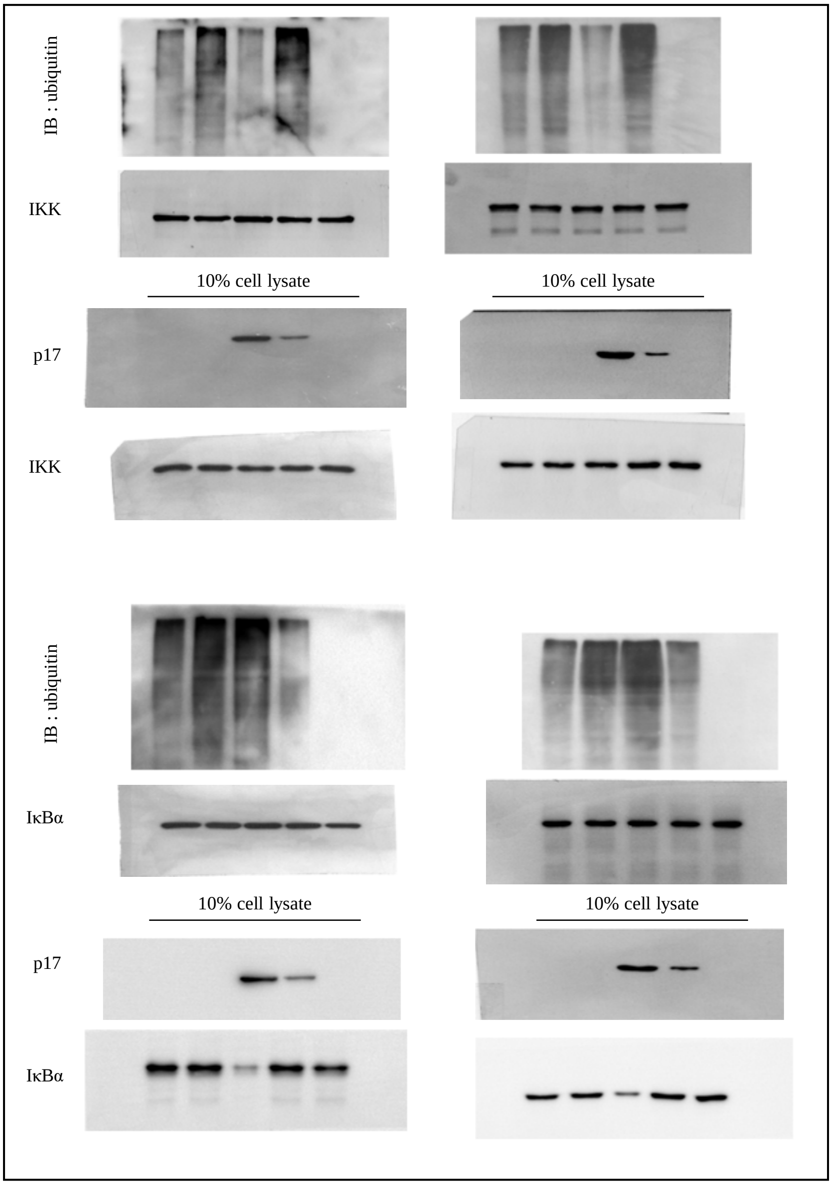

Figure 6A

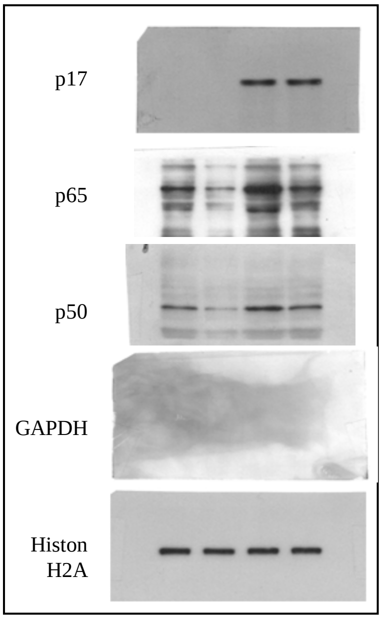

Figure 6B

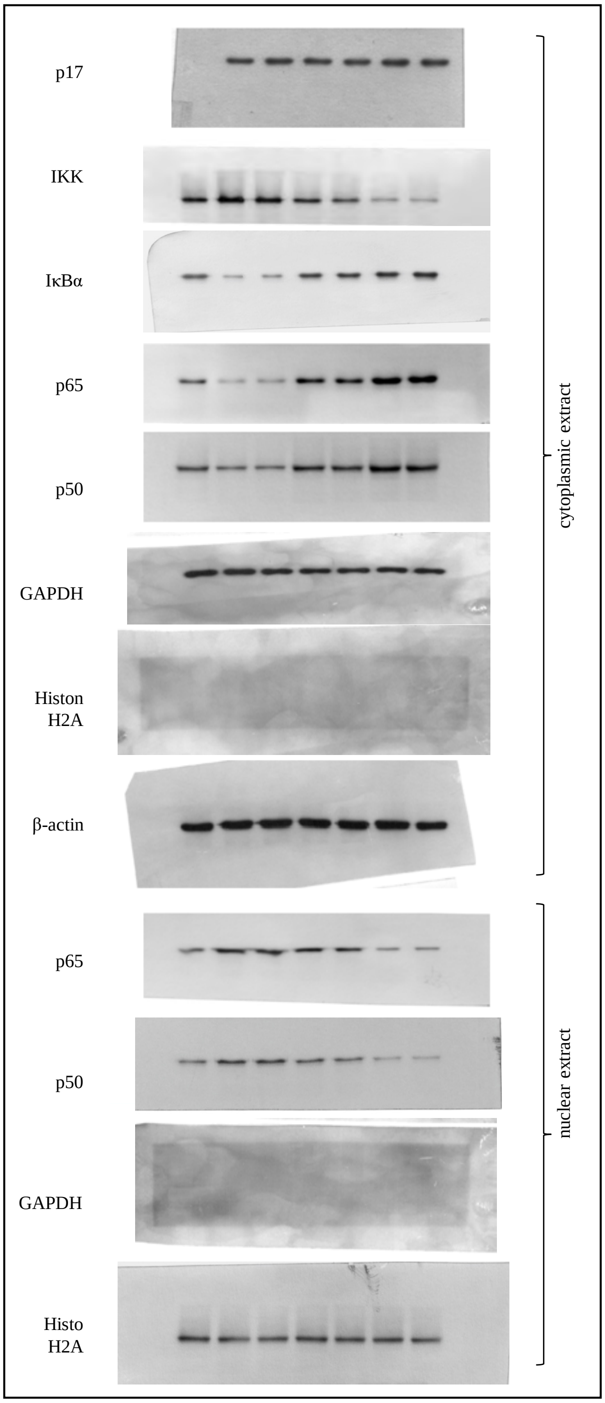

Figure 6D

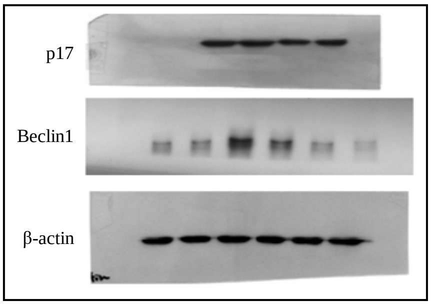

Figure 7A

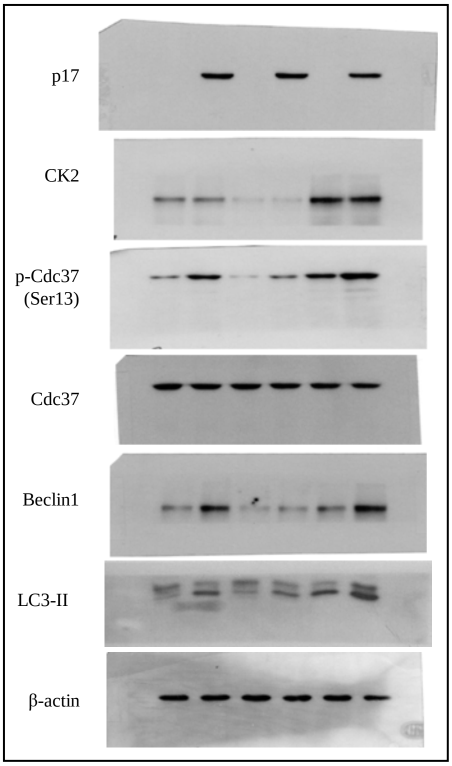

Figure 7B

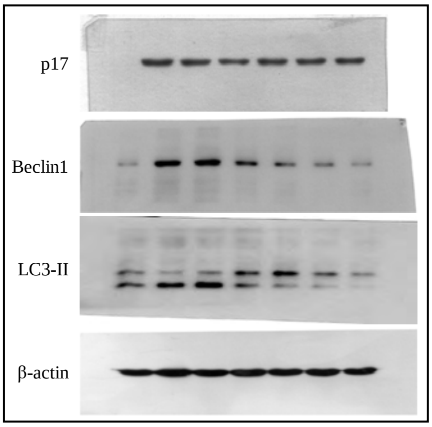

Figure 7C

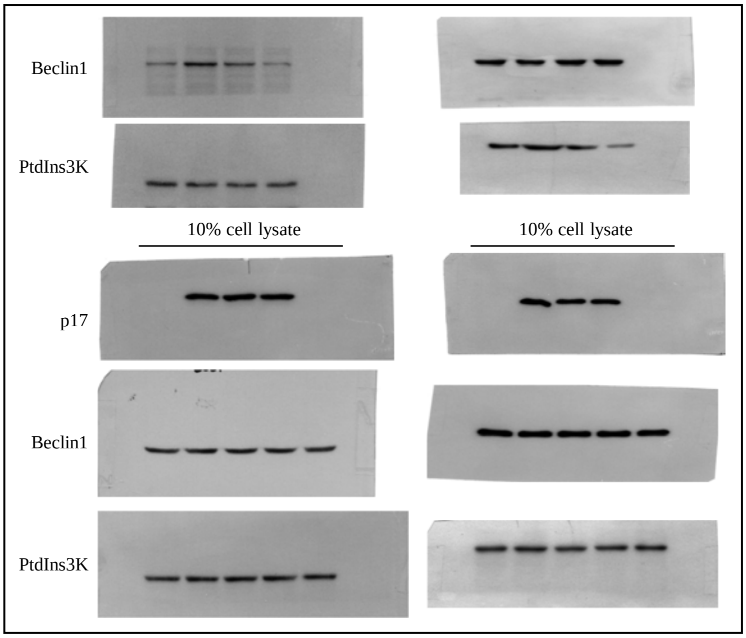

Figure 9A

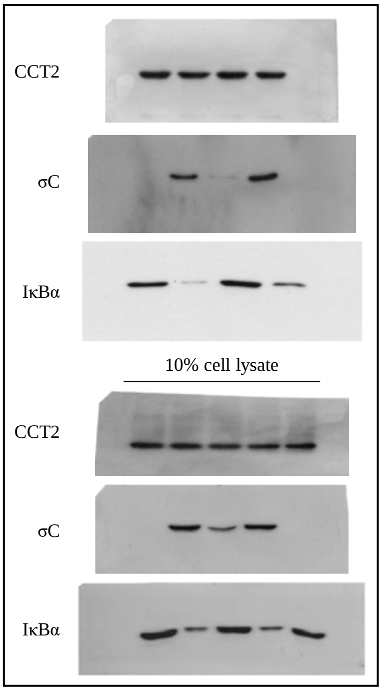

Figure 9B

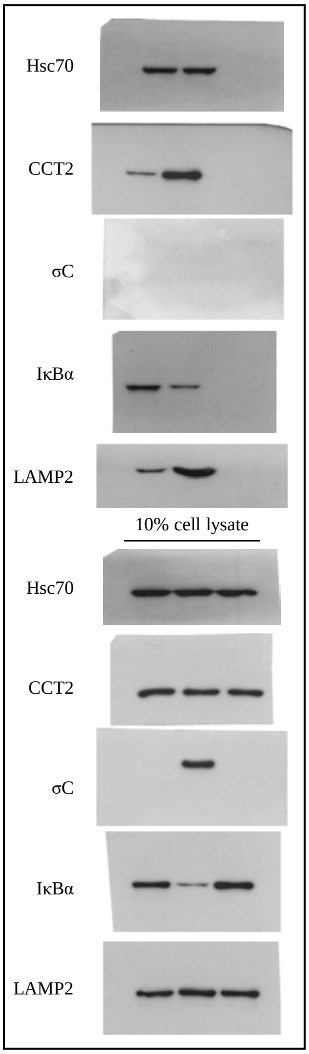

Figure 8A

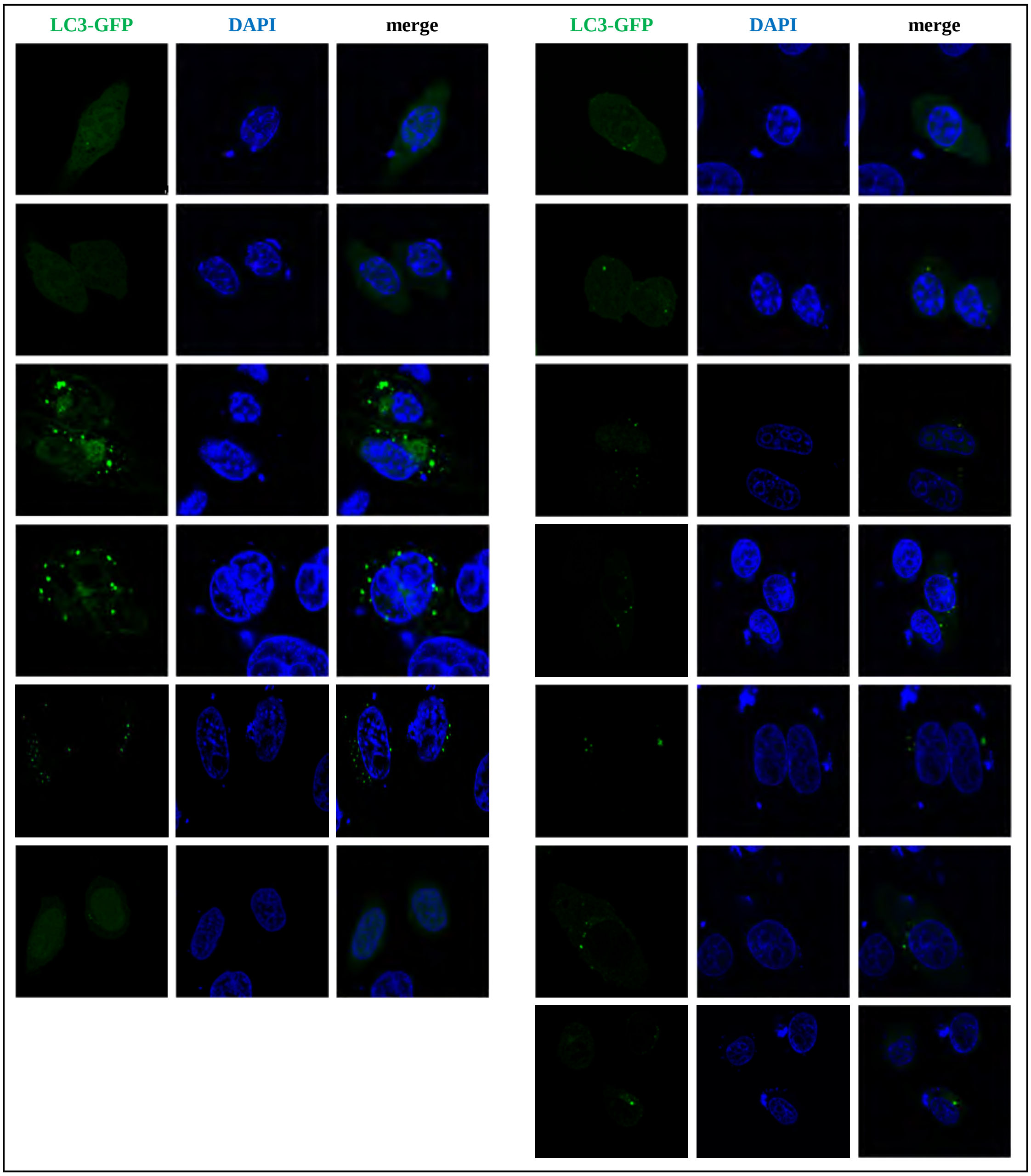

Figure 9C

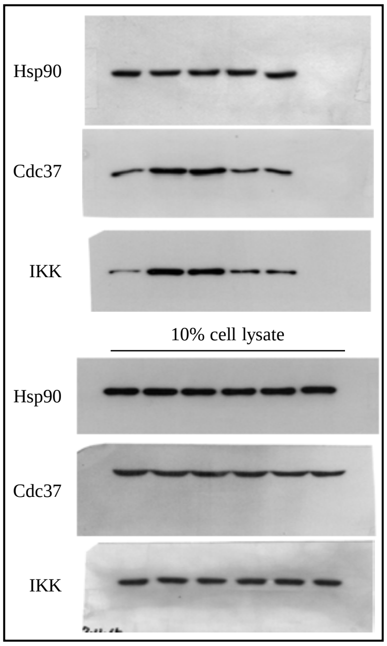

Figure 9D

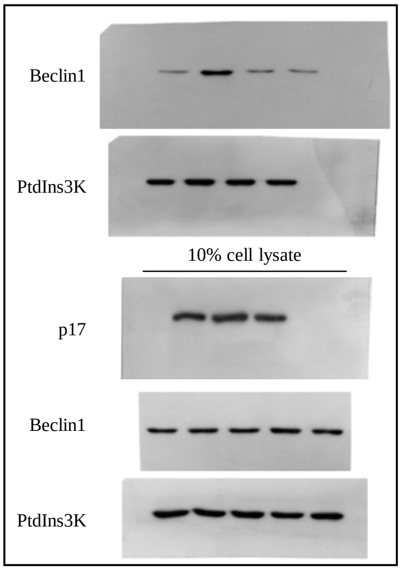

Figure S1F

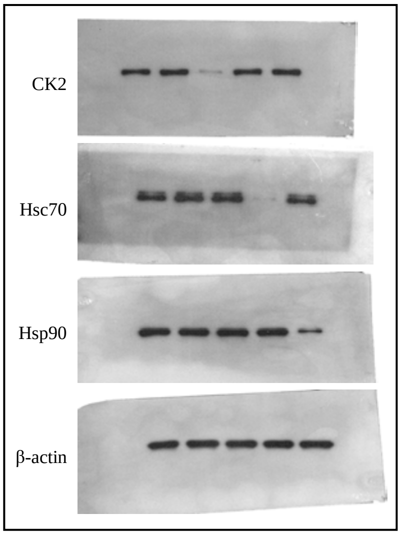

Figure 9E

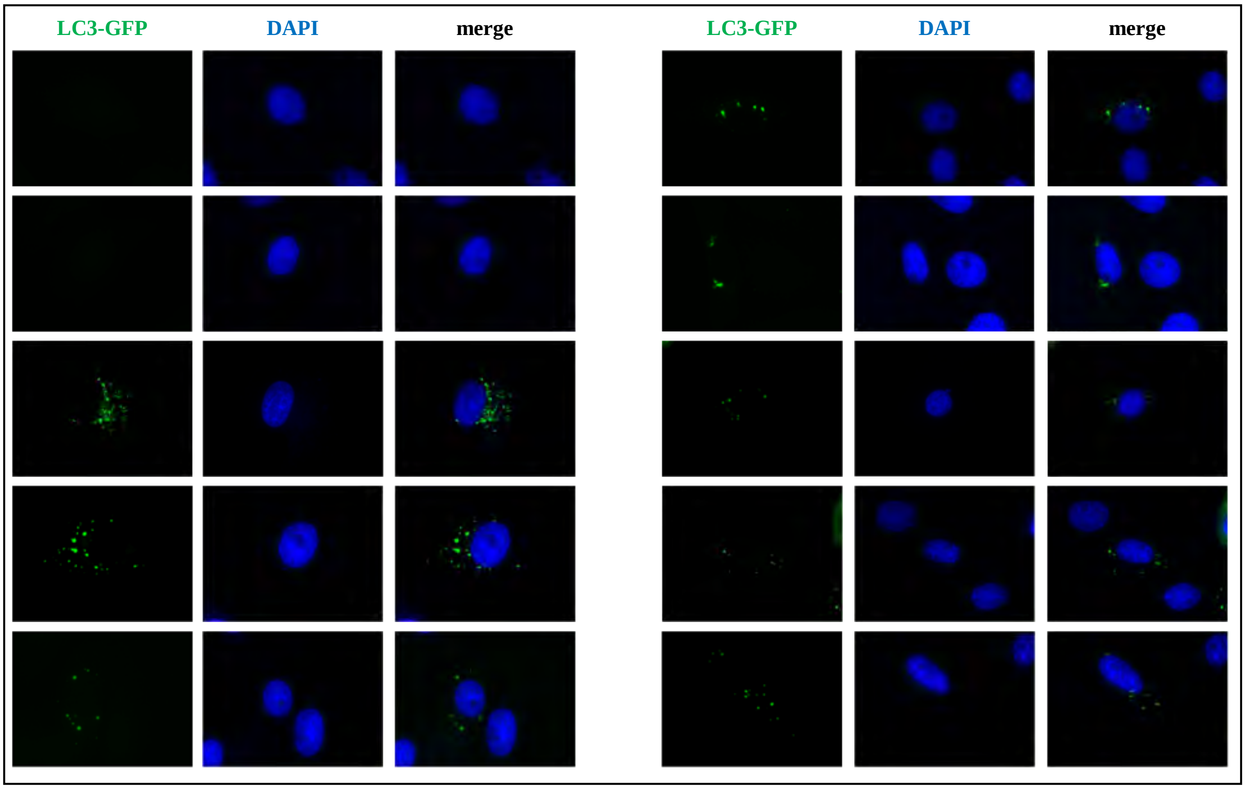

Supplement: Supplemental figures — Figures S1 to S7. [file jvi.01089-25-s0001.pdf]
